# Criteria for the Characterization of Seafood Byproducts to Allow Tracing Their Geographic Origin

**DOI:** 10.3390/foods15061073

**Published:** 2026-03-18

**Authors:** Cláudia P. Passos, Fernando Ricardo, Ricardo Calado

**Affiliations:** 1 LAQV-REQUIMTE, Departamento de Química, Universidade de Aveiro, Campus Universitário de Santiago, 3010-193 Aveiro, Portugal; 2 ECOMARE, CESAM, Departamento de Biologia, Universidade de Aveiro, Campus Universitário de Santiago, 3810-193 Aveiro, Portugal; fafr@ua.pt

**Keywords:** marine bioresources, authenticity, traceability, valorisation, collagen, hydroxyapatite, chitosan

## Abstract

Marine byproducts generated from seafood processing represent valuable reservoirs of structurally and functionally distinct biomolecules, whose composition reflects species, habitat, and processing history. This systematic review identified which marine byproducts have been most extensively studied between 2020 and 2025, with emphasis on their composition, valorisation, and suitability for tracing their geographic origin. Following the PRISMA protocol, 6443 publications were initially retrieved, of which 96 peer-reviewed studies were included for data extraction and analysis. The five most frequently investigated byproducts—skin, bones, scales, shells, and roe—were identified as rich sources of proteins (collagen and gelatin), minerals (hydroxyapatite and calcium carbonate), polysaccharides (chitin), lipids (notably polyunsaturated fatty acids (PUFAs), docosahexaenoic acid (DHA) and eicosapentaenoic acid (EPA)), and vitamin B12. Collagen properties, particularly imino acid content, hydroxylation degree, crosslinking density, and thermal stability, correlate more strongly with environmental temperature than taxonomy, supporting their potential as markers for tracing geographic origin. The mineral fractions, dominated by hydroxyapatite in bones and scales, or calcium carbonate in shells, provided complementary inorganic fingerprints based on calcium-to-phosphorus ratios, carbonate substitution, trace element composition, and thermal analyses. While the lipid profile alone could not completely discriminate fish roe, proteomic techniques, such as MALDI-TOF MS, make it possible to reliably identify species. Collectively, these byproducts offer complementary organic and inorganic markers that support integrated strategies that allow tracing their origin and fostering their sustainable valorisation, overcoming a key technical bottleneck for their use. However, their large-scale conversion into market-ready products remains limited by technical complexity, process variability, and cost-related constraints.

## 1. Introduction

The growth of the global population has led to a substantial increase in food demand. In parallel, global fish production has risen markedly, from 19 million tonnes (Mt) in the early 1950s [1] to 160 Mt in 2010, 214 Mt in 2020, and 223 Mt in 2022 [2]. Currently, the global production of aquatic animals is almost evenly distributed between capture fisheries (49%) and aquaculture (51%), making it a significant component of the global food industry and a major contributor to fulfilling human nutritional needs [3,4,5,6]. Correspondingly, the apparent annual per capita consumption of aquatic animals increased from 9.1 kg in the 1960s to an estimated 20.7 kg in 2022 [2,7]. In 2021, the United Nations Food and Agriculture Organization (FAO) launched the “Blue Transformation Roadmap”, outlining objectives for the development of aquaculture and fisheries, while emphasising the importance of integrated value chains that ensure social, economic, and environmental sustainability, with the ultimate goal of maximising aquatic food systems. However, the FAO also highlighted in the Blue Transformation Roadmap a lack of reliable traceability systems, which hinders innovation and sustainable trade within value chains and limits the ability to fully ensure product quality, safety, legality, and sustainability [2].

The expansion in production and consumption has been accompanied by a substantial increase in waste generation. The production of fish-related waste is estimated to have risen from 56 Mt in 2010 to 75 Mt in 2020 [6]. At the processing stage, approximately 30–85% of fish catches result in byproducts unsuitable for direct human consumption, including bycatch [4]. These byproducts typically account for 30–40% of the total weight of fresh fish and include heads (~10%), skin (~3%), bones (~15%), viscera (12–18%), and scales (~5%) [4,5,8,9,10,11]. The proportion of byproducts varies according to seafood species and size, as well as fishing season and geographic location [5,12]. In contrast to terrestrial resources, which have achieved recycling rates of approximately 60% from both animal- and plant-based feedstocks, marine bioresources recovery remains comparatively low, with estimates suggesting that only about one-third is currently recovered [6].

Owing to its high microbial load and the activity of endogenous enzymes, seafood deteriorates rapidly if not subjected to appropriate processing and storage conditions, posing significant technological challenges for the food industry [13]. To manage quality and market suitability, many commercially harvested seafood species are classified using a three-level grading system (Extra, A, and B), particularly in industrial-scale fisheries [14]. This system enables sorting based on quality attributes (e.g., size and fat content), freshness indicators (e.g., colour and texture), and intended end use. Species typically subjected to this grading system include Atlantic and Pacific cod, Pollock, Haddock, Hake, Hoki, Whiting and Blue whiting, Mackerel, and Horse mackerel [14]. At the lowest grading level, seafood often exhibits visible defects, discolouration, softer flesh, or early-stage degradation caused by delayed processing, all of which are indicative of incipient decomposition [15]. Consequently, these organisms are frequently diverted from the human food chain and become an additional source of byproducts, sometimes leading to partial or total discarding as waste [16]. Importantly, seafood byproducts require the same stringent processing and storage conditions as whole fish to prevent further deterioration. Despite this requirement, approximately 35% of processed fish is currently classified as “special waste,” a category closely associated with substantial environmental impacts [17]. At present, the predominant uses of seafood byproducts include surimi (fish paste), pet food, fishmeal and low-grade fish oil [2], and fertilisers [17].

Beyond conventional applications, as described above, increasing attention has been directed towards the valorisation of seafood byproducts as renewable natural resources for higher value uses. Their exploitation in pharmaceutical and nutraceutical applications represents a promising strategy for “greening” the chemical product life cycle, while advancing sustainability and environmental protection goals [4]. This shift also requires a reassessment of the term “byproduct” itself. Depending on process adaptability and technological control, materials initially regarded as waste can be transformed into value-added outputs. Under well-established and controlled processing conditions, such materials may generate new revenue streams and be more appropriately defined as “co-products” rather than byproducts [18,19].

Among the most valuable compounds recovered from marine byproducts are biopolymers, notably chitin [12,20] and collagen [3,21]. Chitin is a structurally important polysaccharide and represents the second most abundant biopolymer on Earth after cellulose when total available biomass is considered [22]. For most industrial applications, chitin is deacetylated to produce chitosan, which constitutes the primary commercial derivative [12,20]. Collagen, in contrast, is the main structural protein obtained from both marine and terrestrial sources and can be further processed into gelatin or enzymatically hydrolysed into bioactive peptides [3,8]. Historically, collagen has been sourced predominantly from terrestrial animals, with >90% of commercial gelatin being derived from porcine skin (46%), bovine skin (30%), and bovine bone (24%) collagen hydrolysis [23,24]. However, religious constraints (both Judaism and Islam forbid the consumption of any pork-related products, while Hindus do not consume cow-related products) together with concerns related to zoonotic diseases associated with terrestrial animal sources—such as tuberculosis and bovine spongiform encephalopathy in cattle, and leptospirosis and swine influenza in pigs—have intensified interest in marine-derived alternatives [25]. Although most studies report no incidence of zooanthroponoses associated with seafood [26], the possibility of zoonotic transmission cannot be entirely excluded. Consequently, rigorous processing, hygiene, and safety measures for fish byproducts remain as essential as those applied to terrestrial raw materials.

In addition to safety considerations, allergenicity represents another important factor influencing the utilisation of any source of protein that may be recognised as an allergen, so seafood-derived proteins incorporated into food products must also be carefully assessed in this matter. One effective mitigation strategy involves enzymatic hydrolysis, which converts proteins into shorter peptides, typically with average molecular weights below 3 kDa [7]. These extensively hydrolysed peptides lack antigenic epitopes capable of activating immune responses, thereby substantially reducing allergenic potential [27]. As a result, protein hydrolysates from seafood byproducts are increasingly explored as hypoallergenic ingredients for specialised food formulations and dietary supplements [7,28].

Seafood byproducts also present advantages in the context of specific dietary preferences and religious restrictions, including vegetarian-oriented diets and kosher or halal dietary requirements [25,29]. In halal food systems, seafood is generally permitted; however, certification demands detailed information regarding processing conditions, hygiene standards, and traceability of origin [30,31]. A prominent example of this application is the substitution of mammalian collagen. Collagen, which constitutes approximately 30% of total protein in mammals, is a major component of skin, tendons, connective tissues, and bones [3]. While traditionally extracted from porcine and bovine sources, collagen can also be recovered from seafood byproducts, such as skin, scales, and cartilage. The structure of collagen has been well characterised, with a reported genetic variety of up to 28 types, based on its structure, including fibrous (I, II, III, V and XI) and non-fibrillar (such as IV, VI and VII) [17]. Among fibrillar collagens, which show similar characteristics between marine and human origin, type I collagen (a heterotrimer, consisting of two identical α1 chains (I) and one α2 chain (I)) is the most common [3]. Collagen’s unique structure is attributed to the entanglement of its three polypeptide chains forming a characteristic right-handed triple helix that self-assembles into insoluble fibres of tensile strength [21].

Although marine collagen (as well as in collagen mammal sources) lacks certain essential amino acids and, therefore, cannot serve as a complete nutritional protein, this limitation has not diminished its demand or functional relevance [25,26]. Notably, its lower content of proline and hydroxyproline—key residues governing collagen structure and stability—results in reduced thermal stability [21], but enhanced digestibility [3]. These properties render marine collagen particularly attractive for nutraceutical applications and dietary supplements, especially for consumers seeking alternatives to bovine or porcine products.

Beyond the biopolymers detailed above, fish byproducts are an important source of minerals, including macroelements and trace elements, such as phosphorus (P), potassium (K), calcium (Ca), magnesium (Mg), iron (Fe), zinc (Zn), and strontium (Sr) [3]. Seafood tissues are especially rich in Ca and P, and the Ca:P ratio is frequently reported as a characteristic parameter [17]. Mineral composition is strongly influenced by species, habitat, and feeding regime, suggesting that mineral profiling may serve as a complementary tool for origin traceability when supported by comprehensive reference databases [32].

In parallel with valorisation efforts, food fraud has emerged as a critical issue affecting fisheries and aquaculture products, with implications extending beyond economic losses to encompass public health, social trust, and sustainability [33]. Common fraudulent practices include masking of poor quality, substitution of product constituents, species replacement, mislabelling of geographical origin, repackaging, and tax evasion [34]. These challenges have intensified the demand for rapid, reliable, and cost-effective analytical tools to support fraud detection [33]. Traditional anatomical and morphological analyses are often insufficient due to interspecies similarities and the loss of distinguishing features during the processing of seafood [35]. Consequently, identifying compositional markers within fish byproducts has become a key objective for enhancing traceability.

While advanced analytical approaches, such as DNA barcoding, are sometimes indispensable—particularly for high-value seafood products—their cost and complexity limit their applicability to low-value byproducts. Therefore, the development of simpler, more accessible analytical methodologies, based on more affordable technologies, is essential to enable routine verification and strengthen confidence across seafood value chains.

In the present review, we identify which marine organisms that are either captured or farmed (e.g., tuna, cod, salmon, and shrimp) more strongly contribute to the production of seafood. Subsequently, we surveyed the scientific literature to identify which were the most prevalent byproducts produced (e.g., skin, scales, bones, shell, roe) and relate them to the world distribution of the species from which they are derived (e.g., cold-water, temperate or tropical marine species) to better understand how these can shape their composition. Finally, each of these seafood byproducts was examined based on its intrinsic features and compositional richness to identify features that can be used for tracing their geographic origin.

## 2. Materials and Methods

### 2.1. Search Strategies

The search strategy applied in this systematic review was designed to address the following research question: *Can strategies be defined to trace the origin of marine byproducts with valorisation potential?* To systematically answer this question, three core conceptual domains were identified for keyword development: Concept 1, *origin traceability*; Concept 2, *marine byproducts*; and Concept 3, *valorisation*.

**Figure 1:**
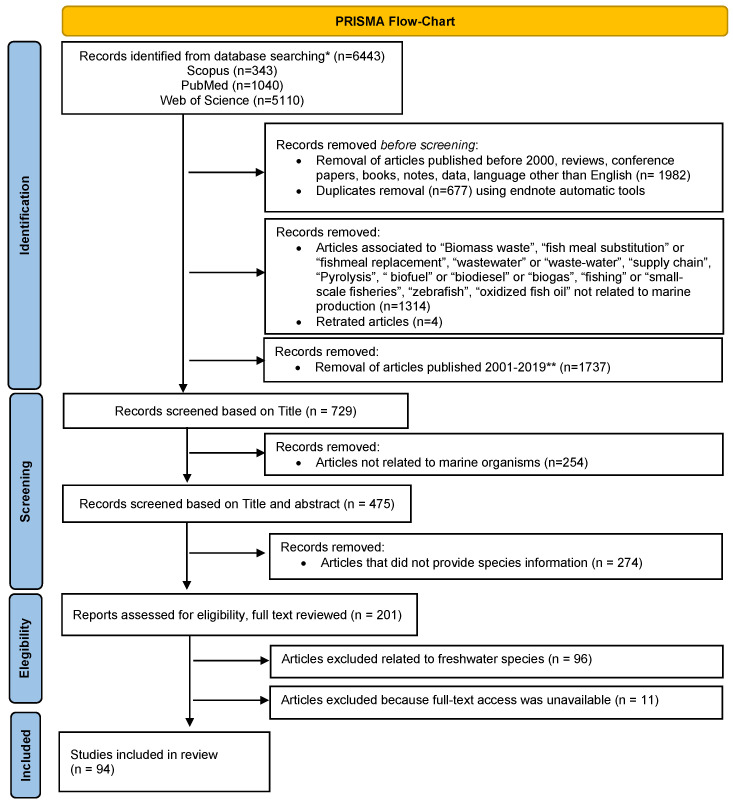
Scheme of the adopted PRISMA flow-chart. * The literature search was performed using combinations of keywords derived from these concepts, specifically pairing *origin traceability* with *marine byproducts*, and *marine byproducts* with *valorisation*. ** Due to the large number of publications retrieved, the screening was restricted to the last 5 years (2020–2025).

The literature search was performed using combinations of keywords derived from these concepts, specifically pairing *origin traceability* with *marine byproducts* and *marine byproducts* with *valorisation*. A detailed description of the keywords and search strings employed is provided in the Supplementary Materials. The article collection included publications available up to 20 August 2025. The study selection process followed the PRISMA guidelines, and the corresponding PRISMA flowchart summarising the screening and inclusion steps is presented in Figure 1.

A total of 6443 publications were initially identified through searches of three bibliographic databases: Scopus, PubMed, and Web of Science. Publications released prior to 2000, as well as review articles, conference proceedings, books, and articles written in languages other than English, were excluded. All retrieved records were imported into an EndNote library, where duplicate entries were identified and removed.

A preliminary screening of article titles from a subset of the records retrieved enabled the identification of misleading or non-relevant keywords, including “biomass waste,” “fish meal substitution” or “fishmeal replacement,” “wastewater” or “waste-water,” “supply chain,” “pyrolysis,” “biofuel,” “biodiesel,” “biogas,” “fishing” or “small-scale fisheries,” “zebrafish,” and “oxidized fish oil,” which were considered to be outside the scope of the present review. Retracted articles were also excluded at this stage. Despite the exclusions, the number of remaining records was still substantial. Therefore, the search criteria were further restricted to publications from the last five years, yielding a final dataset of 729 articles. Subsequent screening based on titles and abstracts identified 201 articles that reported information on seafood byproducts and corresponding species, which were then subjected to a full-text review.

Following full-text assessment, only 94 articles met the eligibility criteria by providing explicit information on marine byproducts with clear species identification and were therefore selected for data extraction [1,3,4,5,6,7,8,9,10,11,12,16,17,20,21,23,24,28,36,37,38,39,40,41,42,43,44,45,46,47,48,49,50,51,52,53,54,55,56,57,58,59,60,61,62,63,64,65,66,67,68,69,70,71,72,73,74,75,76,77,78,79,80,81,82,83,84,85,86,87,88,89,90,91,92,93,94,95,96,97,98,99,100,101,102,103,104,105,106,107,108,109,110,111,112,113].

### 2.2. Exclusion Criteria Explained

The 729 articles identified after database filtering were screened based on title information, and records not related to the marine context were excluded. Given that the primary objective of this review was to investigate whether and how it is possible to trace the origin of marine byproducts, studies that did not provide explicit information on the species being addressed were excluded. Consequently, of the 475 articles initially remaining after removing those that did not address marine organisms, an additional 274 articles were also excluded for not detailing the seafood species being studied. During the eligibility phase, two authors independently reviewed the titles and abstracts of the remaining 201 articles. Studies identified as focusing exclusively on freshwater species were once more excluded, as they fell outside the scope of the present review. In addition, articles for which full-text access was unavailable (n = 11) were removed from the dataset. The remaining 94 articles were subsequently assessed through full-text review.

At all stages of the selection process, any disagreements between the two reviewers were resolved through discussion until consensus was reached, and articles were either included or excluded in accordance with the predefined eligibility criteria.

### 2.3. Data Processing

The Alluvial diagrams presented in this manuscript were generated with the open-source software RAWGraphs 2.0, an open-source data visualisation framework project, led and maintained by the DensityDesign Research Lab (Politecnico di Milano) with the goal of making the visual representation of complex data easy for everyone (https://www.rawgraphs.io/).

## 3. Results and Discussion

### 3.1. Global Data Analysis for Captured and Farmed Seafood and Relevant Case Studies

Before discussing the literature survey on marine byproducts derived from seafood processing, this section presents a global data analysis of fish production, recognising that byproducts originate from both wild-caught fisheries and aquaculture, and their corresponding processing streams. According to the FAO, food loss and waste occur at all stages of fisheries and aquaculture value chains, including harvesting or farming (and post-catch), processing, distribution (transport, wholesale, and retail), and final consumption [114].

**Figure 2:**
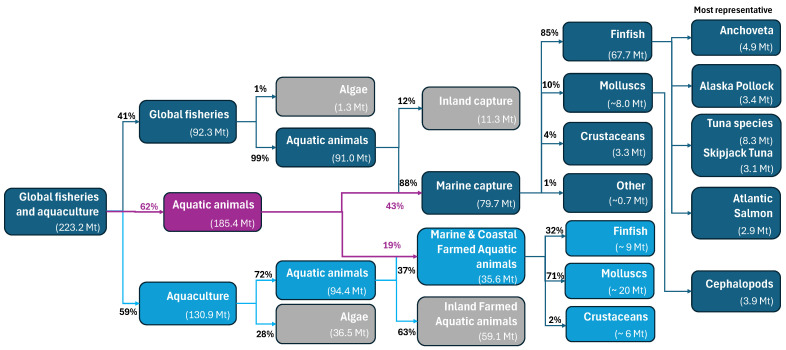
Global fisheries (dark blue line) and aquaculture production (light blue line): data retrieved from the FAO 2024 report [2]. Also represented is the global production of aquatic animals (purple line) with contributions from both wild-caught fisheries and aquaculture. Grey is referenced for algae and aquatic animals from inland capture/production, which are not considered in the present review.

Identifying global production volumes, as presented in Figure 2 for the 2022 fishing season [2], helps direct valorisation efforts toward major production sectors and their associated processing activities, where most recoverable byproducts are generated.

According to FAO data, global fisheries and aquaculture reached a record production of 223.2 Mt in 2022, of which 185.4 Mt corresponded to aquatic animals (purple line, Figure 2). Marine production was distributed between capture fisheries (43%, 79.7 Mt) and aquaculture (31%, 35.6 Mt) [115]. Finfish—aquatic vertebrates with fins and gills and a bony or cartilaginous skeleton (e.g., salmon, tuna, cod)—accounted for approximately 85% of total marine capture production (67.4 Mt). The most representative species were Anchoveta (*Engraulis ringens*, 4.9 Mt), Alaska pollock (*Gadus chalcogrammus*, 3.4 Mt), Skipjack tuna (*Katsuwonus pelamis*, 3.1 Mt), and Atlantic salmon (*Salmo salar*, 2.9 Mt). Additionally, cephalopods (3.9 Mt) and crustaceans (3.3 Mt), mainly shrimp and lobsters, made substantial contributions to capture fisheries [115]. Within marine and coastal farmed aquaculture, finfish accounted for approximately 32% of total marine production, crustaceans for about 2%, and molluscs represented the largest share (approximately 71%) (Figure 2).

The contribution of different value-chain stages to food loss and waste varies considerably by region. In highly developed regions, such as Europe, North America, and Oceania, processing is among the least significant contributors, largely because byproducts are recovered to a greater extent than in other sectors [114]. Consequently, marine processing plants provide one of the most reliable estimates of effective byproduct utilisation, especially considering that about 70% of the total catch is processed [116]. This observation is particularly relevant in high-income countries, where aquatic foods are predominantly consumed in processed form. Recent FAO reports indicate that 34% of fishmeal and 53% of fish oil are now produced from fish byproducts, reflecting a substantial shift toward circular resource use [2].

Industrial examples demonstrating the feasibility and benefits of maximising byproduct utilisation are increasingly emerging. Based on the global production distribution shown in Figure 2, selected case studies of major relevance to marine fisheries and aquaculture are presented below.

#### 3.1.1. Case Study: Wild-Caught Fisheries—Tuna (*Thunnus* spp.)

Tuna represents one of the most commercially important marine fish groups and is widely distributed in tropical waters—such as skipjack (*Katsuwonus pelamis*), yellowfin (*Thunnus albacares*), and bigeye (*Thunnus obesus*)—as well as temperate waters, including albacore (*Thunnus alalunga*) and bluefin species (*Thunnus thynnus*, *Thunnus orientalis*, and *Thunnus maccoyii*). Global catches of the principal tuna market species increased from 4.3 Mt in 2010 [117] to 8.3 Mt in 2022, with skipjack tuna (*Katsuwonus pelamis*) alone accounting for 3.1 Mt (Figure 2).

Tuna is primarily marketed fresh, chilled, frozen, or canned. Canning and loin processing, which represent the main industrial processing routes for tuna, generate substantial amounts of byproducts, accounting for up to 70% of the original biomass [7]. These include belly flaps, off-cut meat, bone-adhered mince, blood meat, heads, viscera, tails, skin, and bones [1,5,117]. Most solid tuna-processing residues—particularly heads, fins, bones, and dark (red) meat (this last one representing approximately 9–11% of total body weight)—are currently commonly converted into fishmeal for animal feed [117]. Despite its high protein content, dark meat has limited applications due to its colour, susceptibility to oxidation, and off-flavour development [117]. An additional waste stream, corresponding to 5–10% of initial tuna weight, consists of meat fragments that are undersized, oversized, or deformed for canning [7]. Processing operations such as cooking induce protein denaturation, leading to reduced solubility. In addition to solid byproducts, tuna processing generates liquid effluents, including cooking juice (broth), stick water, and washing water, which may contain approximately 4% soluble proteins [117]. These streams can be recovered, concentrated (protein levels exceeding 65% qualify as fish protein concentrates [7]) and further processed into protein hydrolysates.

Indirect valorisation pathways for tuna byproducts include silage production, obtained by acidification of whole fish or fish parts, enabling enzymatic liquefaction by endogenous enzymes [118]. Fish silage is primarily used as an alternative to the production of fishmeal, for both terrestrial and aquaculture feeds. Tuna viscera also represent a valuable source of digestive enzymes, while tuna bones may be processed into calcium-rich materials following deproteinization and lipid removal [117].

#### 3.1.2. Case Study: Wild-Caught Fisheries—Icelandic Cod (*Gadus morhua*)

A prominent example of high-value byproduct valorisation within the blue bioeconomy is provided by the Iceland Ocean Cluster, which established the Codland initiative. This private-sector-driven model aims to maximise value extraction from all parts of wild-caught cod through integrated processing strategies [19]. The initiative emerged in response to strict catch limitations, which limited revenue and necessitated maximising value rather than volume. As a result, the economic value of cod catches reportedly increased by approximately 40% [119].

A key success factor was the co-location of processing facilities near ports and cod-drying plants, minimising transportation costs and enabling efficient side-stream utilisation. The processing approach relies heavily on biotechnological methods, including enzymatic hydrolysis to recover collagen and partially hydrolysed collagen peptides [19]. The overarching objective is full (100%) utilisation of all byproducts, including liver- and viscera-derived fish oil, dried products, and fishmeal [19]. End-use applications span agriculture (organic fertilisers), food and agro-industry (omega-3 supplements and nutraceuticals), healthcare (calcium-based mineral supplements), and biopharmaceutical sectors (collagen-derived peptides for skin-care applications) [119].

#### 3.1.3. Case Study: Aquaculture—*Atlantic salmon* (*Salmo salar*)

Norway was among the first countries to recognise the economic and environmental value of seafood byproducts. Currently, the Norwegian Atlantic salmon industry processes over 1.5 Mt annually (the world’s largest producer with ~50% of global production), primarily from farmed Atlantic salmon (*Salmo salar*), and recovers approximately 90% of all byproducts it generates [119,120]. Chile, the world’s second-largest salmon producer (about half the volume of Norway), reports similarly high recovery rates; however, most Chilean byproducts are still converted into lower-value products, such as fishmeal and fish oil [105]. Although higher-value applications require greater capital investment, their market prices can outweigh initial costs.

One standardised valorisation pathway in Norway is silage production through acid-enzymatic hydrolysis, primarily used for animal feed, including for fur-bearing animals [121]. More technologically advanced processes involve the extraction of collagen, gelatin, and their hydrolysates. Despite existing constraints, industrial-scale implementations of such technologies are reported in regions including Alaska, Scotland, and Norway (e.g., Bergen) [121]. In contrast, the Chilean salmon sector faces ongoing challenges related to environmental, health, and logistical aspects of byproduct management [121].

**Figure 3:**
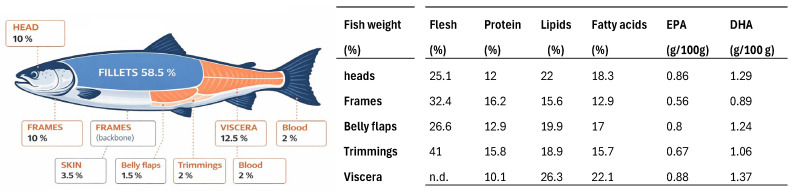
*Atlantic salmon* (*Salmo salar*) processing byproducts (% of whole fish weight) and byproduct distribution and nutritional analysis [119]. Figure elements were assembled from [119] and refined with assistance from ChatGPT (OpenAI), with all scientific content being confirmed by the authors. n.d.—not determined.

Farmed salmon are harvested, slaughtered, and bled before undergoing primary processing to produce head-on gutted fish. Secondary processing generates trimmings (2%), heads (10%), frames (10%), skin (3.5%), and belly flaps (1.5%), with these byproducts being schematically represented in Figure 3 [116,119]. FAO reports indicate that salmon byproducts may display high nutritional value, featuring levels of valuable fatty acids (e.g., DHA) comparable to those present in fillets [119].

In automated filleting operations, fillets represent approximately 59–63% of body weight, while heads (10–12%) and spines (9–15%) are generated as byproducts [11,113]. Although most salmon byproducts are recovered, the economic return depends strongly on the technological investments made.

Theoretical modelling of Atlantic salmon (*Salmo salar*) aquaculture suggests that strategic byproduct management could increase food production by up to 60% from the same farming output, corresponding to an 803% revenue increase derived from byproduct utilisation alone [119]. Valorisation pathways include applications in agriculture (10% for fuel and organic fertilisers) and predominantly food and feed sectors, distributed among human consumption (15%), pet food (22%), livestock feed (46%), and aquafeed (7%) [119]. While many applications rely on mechanical processing (e.g., fishmeal and oil production), advanced technologies, such as protein hydrolysis for collagen and peptide recovery, play an increasingly important role [44].

#### 3.1.4. Case Study: Marine Coastal Aquaculture—Shrimp

Shrimp byproduct valorisation represents another mature example of near-zero-waste processing, particularly in crustacean industries. Global shrimp production was approximately 3.4 Mt in 2008 [20] and remained relatively stable at around 3.1 Mt in 2022 (Figure 2). Processing typically generates byproducts corresponding to 50–60% of total biomass [122].

In shrimp, the primary byproduct is the cephalothorax (head), which accounts for approximately 45–56% of body weight, depending on species [5,12]. Shrimp are commonly exported frozen, either with or without their exoskeleton (often termed as shells for simplicity), resulting in byproducts composed of heads, viscera, and shells. Many studies do not specify which residues are used for compound recovery; however, the entire cephalothorax is most often processed. As a whole, shrimp heads contain approximately 54% protein, 21% minerals, 12% lipids, 7% chitin, 9% vitamins, and small amounts of carotenoids, particularly astaxanthin [5]. When only the carapace is considered, purified chitin recovery can reach up to 23% (*w*/*w*), approximately three times higher than when processing the entire cephalothorax (e.g., from *Xiphopenaeus kroyeri*) [20].

Similar to tuna processing, shrimp processing generates liquid effluents, such as cooking wastewater, stick water, and washing water, which contain soluble proteins suitable for concentration and conversion into protein hydrolysates [122]. A notable industrial example of shrimp byproduct valorisation is a biorefinery implemented in India, with technological support from the Central Institute of Fisheries Technology, producing chitin, chitosan, and protein hydrolysates for agricultural, pharmaceutical, and cosmetic applications [123].

### 3.2. Literature Survey

Markets for byproducts derived from seafood are increasing, as the desire for these products grows, supported by new technologies able to better use these bioresources. Examples include composting, generation of energy, and greenhouse gas emission reduction [114], which this review has not considered; food and other non-food technological applications are reviewed in the following sections.

**Figure 4:**
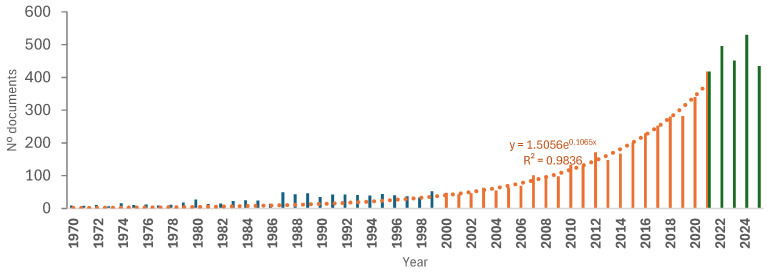
Temporal distribution of publications on seafood byproducts indexed in the Scopus, PubMed, and Web of Science databases using the keyword combinations defined in the systematic review search strategy in accordance with PRISMA guidelines. Publications issued prior to 2000 are represented by blue bars, those published between 2000 and 2020 by orange bars, and those published during the most recent five-year period (2020–2025) by green bars. The exponential growth trend in the number of publications up to 2020 is illustrated by the exponential fit (orange dots), highlighting the marked increase in research activity before stabilization in the most recent five-year period.

From the 6443 publications initially retrieved through searches of three bibliographic databases—Scopus, PubMed, and Web of Science—Figure 4 illustrates the exponential increase in the number of studies addressing seafood byproducts reported over the last half century, up to 2020. In contrast, during the most recent five-year period (2020–2025), this upward trend appears to have stabilized at approximately 500 publications per year.

**Figure 5:**
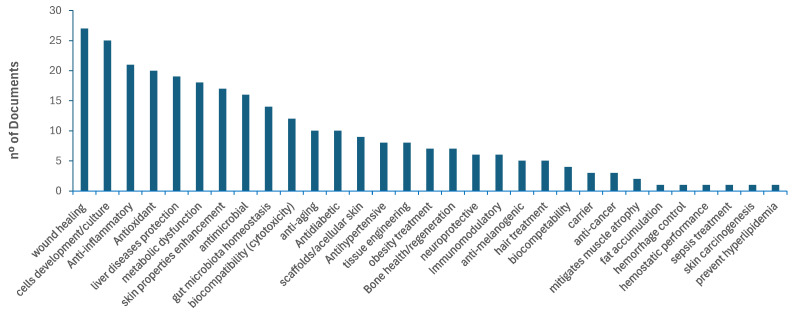
Distribution of scientific articles according to the potential biological activities reported associated with seafood byproducts, as identified in the present review.

**Figure 6:**
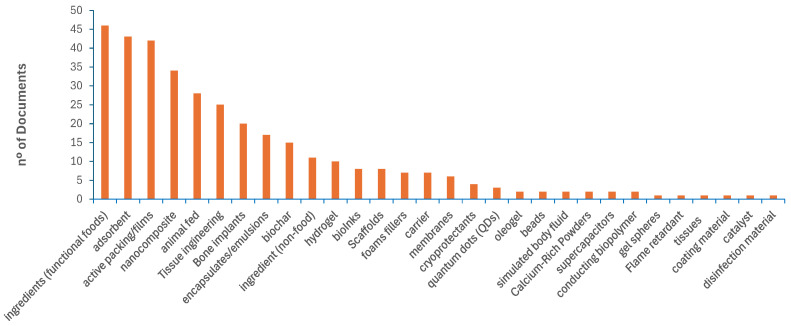
Distribution of scientific articles according to the potential applications reported for seafood byproducts, including food, nutraceutical, pharmaceutical, packaging, biomedical, and other non-food uses, as identified in the present review.

The screening phase, based on titles and abstracts, allowed a simplified overview of literature represented in Figure 5 and Figure 6, respectively showing the corresponding distribution of articles according to the potential biological activities and applications reported, associated with seafood byproducts, as identified in this systematic review.

The FAO has recognised the importance of maximising the utilisation and processing of aquatic products, with food uses accounting for 89% of global aquatic animal production (185.4 Mt) in 2022 [2]. The remaining fraction may be directed toward non-food applications. However, despite the substantial volume of scientific literature on byproducts derived from seafood over recent decades (Figure 4), the wide range of bioactivities reported associated with their major components (Figure 5), and the diversity of potential applications described in the literature (Figure 6), most commercially relevant uses of targeting these byproducts remain largely confined to fishmeal and fish oil production [2].

The extraction of biologically active compounds—such as proteins [9], bioactive peptides [4,41,105], carbohydrates [12], fatty acids [66], and minerals [3]—offers significant potential to reduce waste, while also making it possible to increase the availability of value-added ingredients for human consumption, either as food or functional extracts. The identification of health-related bioactivities in seafood byproducts has become increasingly prominent in the literature, as illustrated in Figure 5. These activities have been reported for minimally processed byproducts (e.g., liver oil [107] and fish oil [48]), crude extracts (e.g., lipidic and aqueous extracts [98,124]), enriched fractions such as protein hydrolysates [4,8,95,111,113,125], and purified individual components, including collagen [21] and hydroxyapatite [57,101,108]. Among the most frequently reported health-related bioactivities are antimicrobial (including antibacterial, antifungal, and antiviral) effects [1,93,107], antioxidant activity [7,8], antihypertensive effects [8,95], immunomodulatory activity [49], skin anti-ageing potential [126], anti-obesity effects [79,87], and antidiabetic activity [24,56]. Collectively, these findings highlight the growing relevance of marine byproducts as sources of functional and bioactive compounds.

When food-related applications are not feasible, marine byproducts may also be directed toward non-food uses. These include applications in packaging materials [12,23,36], tissue engineering [57,101,108], wound healing [51,99,101], nanocomposites [101], and adsorbent materials [38,71,104,108,110], among others. Examples of the most frequently reported applications in the literature over the last five years (2020–2025) are summarised in Figure 6. Notably, some approaches combine multiple byproduct-derived components to enhance functionality, such as the synergistic use of gelatin matrices reinforced with nanohydroxyapatite to improve mechanical and adsorptive properties [45] or the development of composite films based on collagen combined with chitosan to enhance material performance [9].

The following section presents information retrieved from the full-text assessment of the 94 articles that fulfil all eligibility criteria by providing explicit information on marine byproducts with a clear identification of the species being addressed, thus allowing successful data extraction.

### 3.3. Byproducts’ Prevalence and Composition

Fish-processing waste can be broadly categorised into highly perishable byproducts (e.g., viscera and blood) and relatively stable byproducts (e.g., bones, heads, scales, and skin) [4]. This distinction is primarily associated with differences in moisture content and biochemical composition. Stable byproducts generally exhibit lower moisture levels and reduced enzymatic activity, which limits microbial growth and delays spoilage [5]. When multiple byproducts are combined, additional stabilisation treatments, such as drying, have been proposed to preserve matrix integrity and prevent degradation [7]. Shell-derived byproducts are typically more stable than viscera or blood but contain higher moisture levels than bones or scales and, therefore, require careful handling. The presence of chitin in shrimp and crab shells contributes to their hydrophilic properties, which increase water retention and influences the storage stability of the derived products [5].

**Figure 7:**
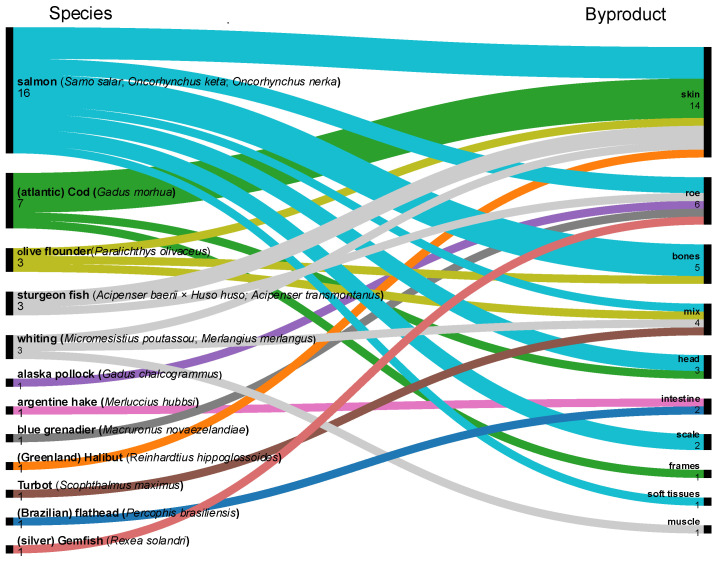
Distribution of cold-water marine fish species (polar, subpolar, boreal, and cold-temperate regions) by number of documents, in relation to the byproducts addressed (skin, roe, bones, mixed byproducts, intestine, scales, head, frames, soft tissues, and muscle). The width of each category reflects the number of publications and the strength of connections between species and byproduct types. When more than one subspecies was reported, all identified taxa are represented.

**Figure 8:**
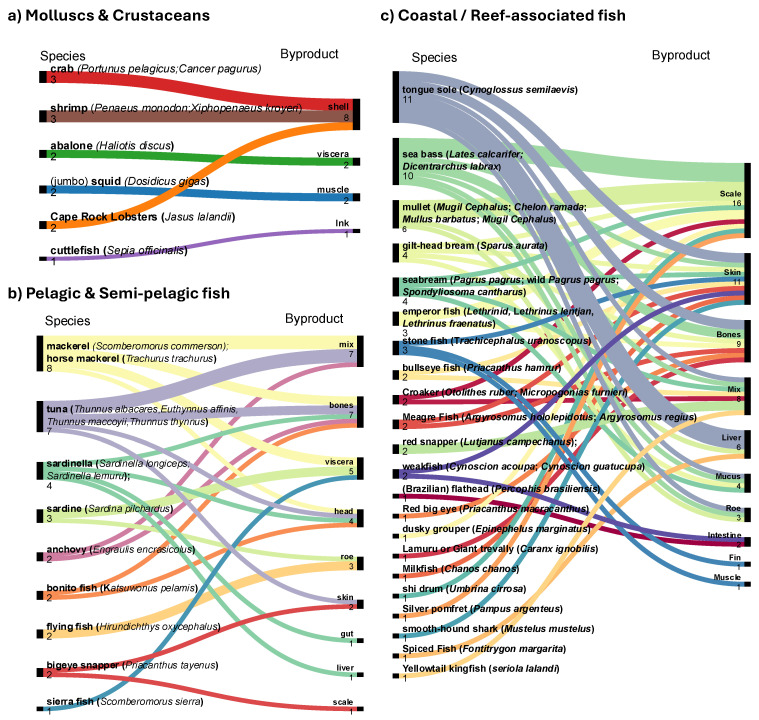
Distribution of temperate and tropical marine species by number of documents, in relation to the byproducts addressed (shells, viscera, muscle, ink, mixed byproducts, bones, heads, roe, gut, liver, skin, scales, intestine, frames, mucus, and fins). The width of each category reflects publication frequency and connectivity between species and byproduct types. Subfigures represent: (**a**) molluscs and crustaceans; (**b**) pelagic and semi-pelagic fish; and (**c**) coastal and reef-associated fish. When available, subspecies information is included.

Based on data extracted from the 94 articles identified through the PRISMA screening process, the marine byproducts most frequently reported in the literature across all species (Figure 7 and Figure 8) were, in descending order of publication frequency: skin (26 documents), bones (21), scales (18), mixed byproducts (19; undefined combinations of multiple tissues), roe (12), shells (8), viscera (7), heads (7), liver (7), mucus (4), muscle (4), intestine (3), frames (1), ink (1), gut (1), soft tissues (1), and fins (1).

Figure 7 and Figure 8 summarise the distribution of byproducts across 50 identified marine species, categorised according to their thermal habitat. Cold-water species inhabiting polar, subpolar, boreal, and cold-temperate regions are presented in Figure 7. Temperate and tropical species are shown in Figure 8, further subdivided into (a) molluscs and crustaceans, (b) pelagic and semi-pelagic fish, and (c) coastal and reef-associated fish. When available, information on subspecies is included to reflect taxonomic diversity.

**Figure 9:**
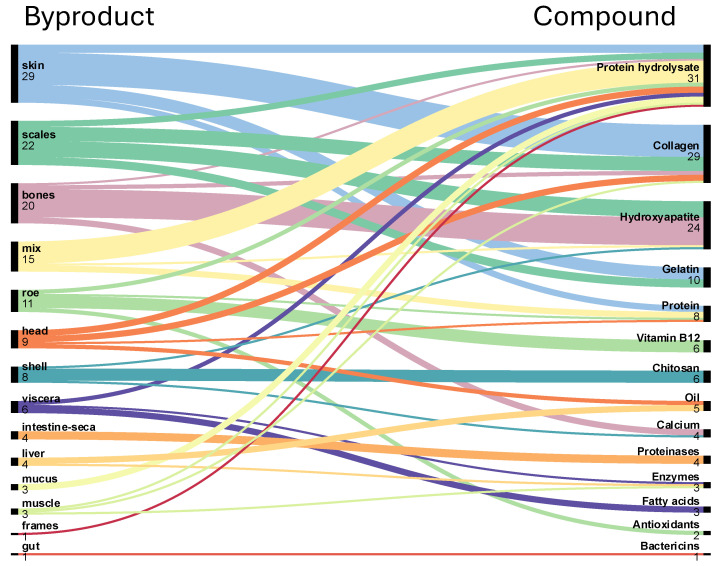
Correlation between seafood byproducts (skin, scales, bones, roe, heads, shells, viscera, intestine, liver, mucus, muscle, frames, and gut) and extracted bioactive compounds of interest, including collagen, gelatin, protein hydrolysates and proteins (generic), hydroxyapatite, vitamin B12, chitosan, oils, enzymes (particularly proteinases), calcium, fatty acids, antioxidants, and bacteriocins. The width of each category reflects the number of publications and the strength of associations between byproducts and bioactive compounds.

In addition, seafood byproducts were correlated with the bioactive compounds extracted from them (Figure 9). Based on frequency and relevance, the five most cited byproducts—skin, bones, scales, roe, and shells—were selected for detailed discussion in subsequent sections. The most common analytical methods used to characterise these compounds are listed in Table 1. Mixed byproducts were excluded from this analysis due to their heterogeneous composition and limited interpretability.

#### 3.3.1. Skin, Scales, and Bones as Sources of Collagen

Fish skin, bones, and scales are among the most important seafood byproducts for protein recovery, particularly collagen [3,8,21]. Collagen extraction from these matrices has been achieved using chemical methods (acidic and/or alkaline treatments) [4,96,97], hydrothermal processes [4], enzymatic approaches, or combinations thereof [127]. Typically, collagen extraction protocols include low-temperature (0–4 °C) pretreatment steps to preserve the native triple-helix structure. These pretreatments commonly involve two sequential operations: (i) deproteinization using dilute alkaline solutions to remove non-collagenous proteins and endogenous proteases, and (ii) demineralisation or decalcification, primarily using dilute acidic solutions [4,96,97,128]. Deproteinization is particularly critical, as endogenous collagenases can degrade collagen during subsequent extraction steps, thereby altering molecular integrity and yield [129]. Demineralisation mainly affects inorganic components such as calcium and is especially relevant for bones and scales. Temperature control is essential, particularly for collagen derived from cold- and temperate-water species (Figure 7), such as cod and salmon, whose collagen denaturation temperature (T_D_) is approximately 15 °C. Consequently, all extraction steps must be performed under strictly controlled low-temperature conditions.

The initial alkaline treatment generally does not solubilise collagen, which remains embedded within connective tissues [4]. Subsequent acid treatment—most commonly with acetic acid, although citric acid has also been reported as an alternative [128]—induces tissue swelling and softening by disrupting non-covalent bonds and allowing solvent penetration into collagen fibres. This process increases collagen solubility and facilitates extraction [56]. Collagen precipitation is commonly achieved through salting-out, followed by centrifugation [128]. Sodium chloride has also been suggested to reduce collagen thermal stability. Extraction becomes more complex in lipid-rich species, such as salmon (Table 1), where lipid–protein aggregates reduce protein digestibility [16] and increase susceptibility to oxidative degradation, potentially causing off-odours [55]. In such cases, defatting steps prior to protein extraction are required [121]. While some studies report that alkaline pretreatment alone is sufficient to remove lipids [129], others include solvent-based defatting (e.g., isopropanol) [55], indicating that protocol optimisation must be species- and matrix-specific. Additional hydrothermal or enzymatic treatments may be applied when gelatin or collagen-derived protein hydrolysates are the target products.

Gelatin is produced through partial hydrolysis and denaturation of collagen. Compared with collagen, gelatin extraction is generally simpler, is less costly, and results in materials with improved stability and processability [17]. Beyond gelatin, shorter protein hydrolysates (PH) derived mainly from collagen disruption have also been obtained, although their characterisation is often less detailed. Hydrolysis destabilises the collagen triple helix by disrupting hydrogen bonds and cross-links [4]. Enzymatic hydrolysis is the most common approach, using proteases such as papain [4], pepsin [21], trypsin, alcalase [16], or pancreatin [65]. However, because enzyme specificity and source matrix strongly influence peptide profiles, comparative interpretation becomes complex; therefore, detailed discussion of enzymatic hydrolysis strategies falls outside the scope of the present review.

Table 2 and Table 3 summarise the proximate composition and amino acid profiles of skin, bone, scale, and cartilage-derived collagen and gelatin from several marine species. Cartilage was considered separately from bone due to compositional differences, but included for comparison, because of similar collagen extraction yields. Representative examples include cold-water species (e.g., cod and salmon, Figure 7) and temperate–tropical species (e.g., bigeye snapper and sharks, Figure 8).

Baltic cod skin (*Gadus morhua callarias*) collagen extracted under acidic conditions (acid-soluble collagen, ASC) [128] was compared with gelatin recovered from Atlantic cod (*Gadus morhua*) after thermal treatment (soluble gelatin, SG) or enzymatic hydrolysis (pancreatin-soluble gelatin, PSG) [65]. Amino acid profiles indicated that extraction severity influenced molecular integrity but not base composition (Table 3). Similarly, salmon (*Salmo salar*) byproducts exhibited higher lipid content (Table 2) than cod (*Gadus morhua*), affecting extraction efficiency and requiring additional defatting steps [121]. Comparisons between skin, bone, and scale collagen revealed matrix-dependent differences in yield and amino acid composition [21,121,129]. Across species, skin consistently yielded higher collagen recovery than bones or scales. For example, collagen extraction yields from bigeye snapper (*Priacanthus tayenus*) reached 10.9% (wet basis) for skin and only 1.6% for bone [130]. Furthermore, normalised compositional parameters, such as hydroxyproline content expressed per gram of protein, minimised methodological bias and highlighted species- and habitat-related differences.

Collagen thermal stability, measured by differential scanning calorimetry (DSC), viscosity, or circular dichroism, is strongly influenced by amino acid composition, particularly imino acid content (proline + hydroxyproline) [127,131]. Collagen denaturation temperature (T_D_) is an irreversible process reflecting triple-helix unwinding, whereas gelatin gelling (T_gel_) and melting temperatures (T_M,gel_) are reversible transitions associated with partial helix reformation [53]. It is important to note that T_D_ can be identified by the temperature at which 50% of collagen molecules are denatured, and viscosity is reduced by half [132]. After gel formation, T_gel_ and T_M,gel_ is related to the physical changes occurring and measured respectively, when cooling or heating the gel. Cold-water species, such as cod (*Gadus morhua*) and Atlantic salmon (*Salmo salar*), exhibit lower T_D_ and gelatin melting temperatures (T_M,gel_) than temperate and tropical species (Table 3), reflecting lower imino acid content. This relationship is well-established and correlates strongly with habitat temperature, rather than taxonomy. For example, sockeye salmon (*Oncorhynchus nerka*) collagen [55] exhibits imino acid contents closer to cod (*Gadus morhua*) than to Atlantic salmon (*Salmo salar*) [129], consistent with colder habitat exposure. In contrast, shark cartilage collagen displays much higher imino acid content and thermal stability, approaching values observed in mammalian collagen, which explains its higher commercial value [133].

Extraction conditions can significantly affect the properties of collagen being recovered. In shark cartilage, differences between acid-soluble (ASC) and pepsin-soluble collagen (PSC) were higher than differences between species (*Carcharhinus limbatus* vs. *Chiloscyllium punctatum*), indicating that extraction methodology may outweigh biological variability [127]. This highlights the importance of standardising protocols when using collagen as a potential origin-tracing marker.

Hydroxyproline hydroxylation also contributes to collagen stability through hydrogen bonding and water-mediated interactions [127]. Differences in hydroxylation degree were observed between species and byproduct types and may serve as additional discriminatory parameters. Lysine hydroxylation (Table 3) further contributes to intermolecular crosslinking and fibril stabilisation. The higher degrees of hydroxylation, particularly in bone-derived collagen, may be justified by a higher requirement for a more stabilised triple helix [130].

Type I collagen is predominant in marine species, although other fibrillar and non-fibrillar collagens also occur (Table 4). Origin tracers were more difficult to identify in gelatin or protein hydrolysates when compared to the original collagen. Beyond amino acid composition, collagen subunit profiles (α, β, and γ chains typically identified with 125 kDa, 250 kDa, and 375 kDa, respectively [129]) provide additional tracing potential. Some literature reports have been able to further distinguish α-1 and α-2 chains [63]; others did not [69]. The short N- and C-terminal regions (called telopeptides) do not form triple helices, since they mainly consist of lysine and hydroxylysine residues and their derivatives [65]. These are linked by both intra- and intermolecular covalent cross-links, thus being important for crosslinking and stabilising the collagen fibril structure [21]. The richness in inter- and intra-molecular cross-linked components, β and γ components respectively, can be used to trace fish-eating habits. Crosslinked β and γ components reflect nutritional status and feeding behaviour, with starved fish exhibiting higher crosslink density, particularly in connective tissues, maintaining the amino acid composition (Table 3), while new collagen is added to the existing one, providing stronger collagen [128,132]. These structural adaptations enhance muscle contraction efficiency during nutrient deprivation.

Analytical variability remains a major limitation in cross-study comparisons. Protein quantification depends strongly on conversion factors (e.g., N × 6.25, N × 5.5, or Hyp × 14.7), and inappropriate molecular weight standards in SDS–PAGE, using globular proteins which do not compensate for the difference from collagenous proteins (which contain a higher content of relatively small amino acids residues (Gly and Ala)), can lead to chain misidentification [17,21,134]. These methodological inconsistencies emphasise the need for normalisation and protocol harmonisation when collagen-based markers are considered for tracing origin. Overall, collagen composition, thermal stability, crosslinking degree, and collagen-type distribution collectively offer strong potential as origin-tracing tools. However, extraction and analytical procedures must be carefully standardised to avoid misleading interpretations.

#### 3.3.2. Scales and Bones as Sources of Hydroxyapatite (HAp) and Calcium

In addition to proteins, seafood byproducts are an important source of minerals, as summarised in Table 5 [3]. The mineral composition of seafood tissues—and consequently of their byproducts—is closely linked to species, habitat, and feeding regime. This variability provides an additional analytical dimension that may be exploited for origin traceability. Unlike skin, which is predominantly proteinaceous (approximately 70% on a dry basis), fish scales and bones exhibit a complex composite structure composed of both organic components (mainly collagen, keratin, and mucin) and inorganic minerals, primarily calcium phosphates [17,41,99,116]. As such, these matrices can be exploited as dual sources of proteins and minerals. In fish scales, organic material represents approximately 30–50% of the total mass [41], while the remaining fraction consists mainly of calcium phosphate compounds, with hydroxyapatite (HAp) accounting for up to 95% of the mineral content [86]. It is noteworthy that, in protein-rich matrices subjected to extensive hydrolysis, the release of free amino acids can induce a salting-out effect, negatively affecting calcium bioaccessibility [3]. Fish bones and scales typically display a low-fat content (Table 5), as most of the fat in fish is concentrated in the flesh rather than the bones.

Fish bones typically consist of approximately 30% organic matter and 70% inorganic material, with HAp comprising around 60–70% of the total mineral fraction [5,69,99]. Bones are separated from the skeletal frame following muscle removal and are subsequently dried and milled to obtain bone powder [5]. This mineral fraction is rich not only in Ca, which exhibits high bioavailability [5], but also in P and Mg, as well as trace elements such as Fe, Zn, and Sr (Table 5).

Calcium phosphates recovered from seafood byproducts can exist primarily as β-tricalcium phosphate (β-TCP; Ca_3_(PO_4_)_2_) or hydroxyapatite (HAp; Ca_10_(PO_4_)_6_(OH)_2_), both of which are widely used as biomaterials. While β-TCP is more resorbable and degrades faster under physiological conditions, HAp is more stable and is commonly applied in bone regeneration and substitution applications [69]. In some biomedical contexts, blends of β-TCP and HAp are employed to balance stability and resorbability [88]. A key descriptor of calcium phosphate materials is the calcium-to-phosphorus atomic ratio (Ca/P), which is 1.5 for β-TCP and 1.67 for stoichiometric HAp. Ca/P ratios lower than 1.67 indicate calcium-deficient hydroxyapatite, which can be converted into β-TCP or HAp under controlled conditions [88,104]. Conversely, elevated Ca/P ratios may indicate non-stoichiometric or defective HAp structures [108]. One such variant is B-type hydroxyapatite, in which carbonate ions (CO_3_^2−^) substitute for phosphate ions (PO_4_^3−^) within the crystal lattice. This substitution results in calcium-deficient HAp, as charge neutrality is maintained by the removal of Ca^2+^ ions [99]. B-type HAp closely resembles biological apatite found in bones and teeth, where carbonate content typically ranges from 5–9% (*w*/*w*) [108]. For example, Ca/P ratios estimated for mullet and seabass scales (Table 5) exceeded typical literature values, suggesting carbonate substitution within the apatite lattice. This interpretation was confirmed by FTIR analysis, which revealed characteristic carbonate absorption bands at 881, 1403, and 1455 cm^−1^ [17,96]. These findings underscore the importance of combining elemental analysis with spectroscopic techniques such as FTIR to accurately characterize mineral phases. Energy-dispersive X-ray (EDX) analysis further supports mineral purity assessment and contaminant detection. In seabass scales, EDX revealed lower Ca/P ratios, consistent with the presence of collagen matrices entrapping apatite crystals [96].

The dual organic–inorganic nature of scales and bones dictates the extraction strategy applied. When hydroxyapatite is the primary target, aggressive deproteinization—often involving acidic treatment at elevated temperatures—is used to remove all organic matter, including collagen. Although HAp is mostly synthesized artificially, it can also be recovered from natural marine sources. Traditional preparation involves high-temperature calcination (800–1000 °C), though alternative low-energy and environmentally friendly methods have recently been proposed [99]. The resulting natural HAp is typically non-stoichiometric and contains trace ions (e.g., Na^+^, Zn^2+^, Mg^2+^, potassium (K^+^), silicon (Si), barium (Ba^2+^), fluoride (F^−^), CO_3_^2−^) that resemble human bone composition and may confer enhanced biological performance [10,99]. These trace elements may also serve as potential tracers for species identification and/or origin.

Thermal analysis techniques, including thermogravimetric analysis (TGA), derivative thermogravimetry (DTG), and differential thermal analysis (DTA), provide valuable compositional fingerprints when combined with chemical characterization. Typical thermal profiles show an initial mass loss at 100–120 °C due to free water evaporation, followed by losses at 130–200 °C associated with chemisorbed water. Organic components decompose mainly between 200 and 500 °C, including processed gelatin [17], and collagen denaturation [96,109], with similar T_max_ ~350 °C. Carbonate decomposition typically occurs between 550 and 800 °C, whereas hydroxyapatite remains thermally stable below 800 °C REF. Bone samples, due to their high mineral content, often require thermal analysis up to 1000 °C [10]. While temperature depends on the nature of the compound, the weight loss at each step reflects the content of each component, such that the final total loss represents a unique signature of the composition. Reported total mass losses vary widely among species, reflecting differences in organic content: 36% for White seabass scales (*Lates calcarifer*) [96], approximately 40% for Atlantic cod (*Gadus morhua*) and Japanese sea bream (*Sparus aurata*) bones, and over 60% for Sardine (*Sardina pilchardus*) bones [10]. Higher losses (60–70%) have been reported for Horse mackerel (*Trachurus trachurus*), tuna, Greater amberjack (*Seriola dumerili*), and Yellowtail or Japanese amberjack (*Seriola quinqueradiata*) bones [135]. Such variability reinforces the potential of thermogravimetric profiles as supplementary tools for tracing the origin of byproducts when combined with compositional data.

#### 3.3.3. Shells as Sources of Chitin and Calcium Carbonate

Compared with fish bones and scales, crustacean shells present a more complex composition due to the presence of a third major component—chitin—strongly interconnected with proteins and mineral fractions [12,20]. Chitin is a linear polysaccharide composed of N-acetyl-D-glucosamine units and can also be sourced from fungi and algae; however, commercial production currently relies almost exclusively on crustacean shell waste from shrimp, crab, lobster, crayfish, and prawn processing [5,20,80]. The primary commercial derivative is chitosan, the deacetylated form of chitin [12,20].

Several biological, enzymatic, and microbial methods have been investigated for chitin extraction [20] to generate chitosan [80]. Nevertheless, chemically based processes remain the most widely adopted to achieve higher yields at lower costs. These processes typically involve sequential steps, namely: demineralization (to remove calcium carbonate), deproteinization, optional decolorization/depigmentation, and deodorization, followed by alkaline deacetylation to produce chitosan [12,20,43]. Deacetylation is commonly performed using concentrated sodium or potassium hydroxide under elevated temperatures, although enzymatic deproteinization steps have also been incorporated in some processes [20,80]. The complexity and severity of chemical processing largely obscure original biological signatures, making it extremely difficult to trace the origin of chitosan based solely on its physicochemical properties. However, complementary information may still be derived from residual mineral components present in shell-derived materials.

Unlike fish bones and scales, crustacean shells are predominantly mineralized with calcium carbonate (CaCO_3_), mainly in the form of calcite or aragonite, and typically do not contain hydroxyapatite. The mineralized CaCO_3_ layer is tightly associated with the epicuticle and outer shell surface, with trace amounts of MgCO_3_ also reported. Mineral content varies by species, reaching approximately 48% in shrimp heads and up to 70% in crab shells [108]. Demineralization processes generate a characteristic porous microstructure by removing mineral phases and opening previously blocked pores [12,42]. This porosity enhances properties, such as ion exchange, adsorption capacity, and potential biomedical performance (e.g., vascularization and bone ingrowth) [108]. Organic components (chitin and proteins) form the remaining matrix, while CaCO_3_ constitutes the dominant inorganic fraction, accounting for 70–90% of total minerals [12,42].

Shell powders can also be subjected to calcination, which transforms CaCO_3_ into CaO or Ca(OH)_2_ [46]. Because carbonate phases are less thermally stable than HAp, lower calcination temperatures (650–700 °C) are typically applied to preserve carbonate content [108]. Some studies further convert calcined shell-derived CaO into HAp via chemical treatment with phosphoric acid [109]; however, such materials should not be considered natural hydroxyapatite. Carbonate-substituted HAp detected in shell-derived materials is generally B-type HAp formed during processing [108].

Thermogravimetric analysis of shrimp shells shows that degradation temperatures and mass-loss patterns are strongly influenced by mineral content. For example, raw shrimp (*Penaeus monodon*) shells exhibited approximately 50% mass loss at 355.9 °C, associated with decomposition of chitin [12], followed by a maximum of 62.6% at 439.0 °C for carbonate decomposition and a total weight loss of 67.23% [12]. Purified chitin and chitosan showed distinct thermal profiles with lower mass loss due to reduced mineral content, but also showed differences from each other, with a maximum of 67.2% (T_max_ 399.7 °C) and 62.6% (T_max_ 409.9 °C), respectively. In the brown crab (*Cancer pagurus*) carapace, TGA revealed a first mass loss (11.3%) at 266–343 °C corresponding to CaCO_3_ decomposition to CaO, followed by a larger loss (39.2%) at 600–740 °C associated with calcite decarboxylation [42]. Activation and processing reduced total mass loss, reflecting increased inorganic content and improved thermal stability [42]. When processing history, organic/inorganic ratios, and species-specific composition are known, such thermal and compositional characteristics may contribute valuable information for tracing the origin of crustacean shell-derived materials.

#### 3.3.4. Roe as a Source of Proteins, PUFAs, and Vitamin B12

Although the terms *eggs* and *roe* are often used interchangeably, a distinction is commonly made between biological and commercial contexts. In biology, *eggs* refer to individual ova, whereas *roe* denotes the collective mass of eggs, a term more frequently used in culinary and commercial applications [136]. Fish roe is also known by different regional names, such as *bottarga* (Italy), *karasumi* (Japan), *caviar* (Russia), and *ikura*, *tarako*, or *tobiko* (Japan) [75,137]. A notable example is flying fish roe, commonly referred to as *tobiko*, which accounts for approximately 20–30% of Indonesia’s total fish roe exports to Asian markets [74].

Historically, fish roe was often regarded as a processing waste. In recent decades, however, it has gained recognition as a high-value food product due to its rich nutritional profile, including high-quality proteins, unsaturated fatty acids, particularly omega-3 PUFAs, water- and fat-soluble vitamins (particularly Vitamin B12), antioxidants (e.g., glutathione and gadusol), and trace elements [66]. Despite this high value, a significant fraction of harvested roe fails to meet premium quality standards. Physical damage caused by rough handling frequently results in rupture of egg sacs, bleeding, discoloration, soft or degraded texture (mushy or watery), and off-odors [16]. In addition, small or underdeveloped eggs are typically classified as low-grade (grade III) roe, which is often diverted to byproduct streams or discarded as waste [16]. Low-grade roe represents an important secondary raw material for the recovery of valuable compounds, particularly proteins, protein hydrolysates, and vitamin B12.

Marine-derived vitamin B12 (Table 6) obtained from roe (can also be obtained from viscera) is of particular interest because it predominantly contains biologically active forms of the vitamin, with only negligible amounts of inactive corrinoids such as pseudo-vitamin B12 or B12 dicarboxylic acids [75]. This represents a significant advantage over alternative sources, such as edible insects, in which up to 95% of extractable B12 may occur in inactive forms. The different contents of vitamin B12 identified in roe from different fish can also be used as a potential tracing tool. Besides the examples presented in Table 6, vitamin B12 has been quantified in white sturgeon (*caviar*, 14.7%), mullet (*bottarga*, 21.6%), and salmon (*sujiko*, 34.7 ± 4.7), this last being different from *Ikura* because it maintains the roe inside the original ovarian sac membrane [75].

From a traceability perspective, morphological identification of fish eggs presents substantial challenges. Early-stage eggs of most marine species exhibit few distinguishing features beyond egg diameter and the size and number of oil droplets in the yolk. Typical egg diameters range from 0.6 to 2.0 mm, with oil droplets measuring approximately 0.1–0.4 mm, a range that overlaps across numerous species [35]. As a result, species identification based solely on morphology is most unreliable. This limitation has fostered the adoption of molecular approaches, such as cytochrome c oxidase subunit I (COI) barcoding, for fish egg identification.

An alternative and increasingly promising technique is matrix-assisted laser desorption/ionisation time-of-flight mass spectrometry (MALDI-TOF MS), which relies on species-specific proteome fingerprints. MALDI-TOF MS has been widely applied in food fraud detection, particularly for seafood species authentication, and has recently been extended to fish egg identification [35]. Compared with DNA barcoding, this technique offers lower operational costs, faster sample preparation, and high identification accuracy [35]. In one study, 210 fish egg samples were initially analysed using COI barcoding, with 178 subsequently validated for a reference library using MALDI-TOF MS. Proteomic clusters derived from mass spectra were congruent with DNA-based groupings, supporting MALDI-TOF MS as a reliable and cost-effective alternative for species identification in ichthyoplankton surveys and traceability studies [35].

Protein-based markers present additional challenges for tracing the origin of roe. Protein content varies not only among species, but also with roe quality grade and seasonality, with lower-grade roe typically exhibiting reduced protein levels [16]. Although proximate composition alone is therefore insufficient for reliable discrimination, electrophoretic techniques such as SDS–PAGE have demonstrated some discriminatory potential. However, high lipid content complicates protein analysis, as lipid–protein aggregates formed through covalent interactions can distort electrophoretic profiles, particularly in low-molecular-weight regions [16,139]. While delipidation steps may partially mitigate this effect, they can also compromise protein integrity, limiting analytical reliability [16]. Furthermore, lipid hydrolysis and oxidation may alter membrane permeability and enzyme activity [140], indirectly influencing protein hydrolysis behaviour [16].

Lipid composition has also been explored as a potential discriminant for roe origin. Comparative studies revealed species-specific differences in fatty acid profiles (Table 6), notably the significantly low contents of EPA (C20:5, *n*-3) and DHA (C22:6, *n*-3) in Kina roe. In all other fish roe, the EPA and DHA together exceed 26%, reaching up to 41% in *Tarako*, the roe from Pollock fish. The relative proportions of saturated fatty acids (SFA), monounsaturated fatty acids (MUFAs), and PUFAs also differed significantly between species. Phospholipid content, particularly phosphatidylcholine (Table 6), also provides statistically robust discrimination.

## 4. Commercial Examples and Future Perspectives

Growing consumer awareness of the unsustainability of linear consumption chains has driven the market towards circular economy approaches, with industry increasingly emphasising the sustainability of production models that utilise byproducts as raw materials [141]. Today, there are already companies that fully use marine byproducts, demonstrating that upcycling is a tangible reality in several marine-based sectors. Table 7 presents high-volume success cases, with commercial products that range from human nutrition to pet food, as well as biofertilizers for agriculture. These examples illustrate how industry is increasingly concerned with resource utilisation, while simultaneously demonstrating how technological solutions can be effectively adapted.

At the same time, the growing presence of these products on the market reflects a shift in consumer perception—from the previously negative association with “waste” towards broader acceptance of byproducts as valuable raw materials in new product lines [142]. This new approach towards seafood by-products highlights the need to develop suitable traceability pathways and validate which existing protocols for seafood in general can be replicated.

**Table 7:** Examples of companies that have a high impact on marine byproduct utilisation and their commercial products.

Company/ Marine Byproducts (Estimated Utilisation)	Commercial Products
Biomega Group [143] (Norway) Salmon byproducts (~36,000 t/year)	Human Nutrition Ingredients: SalMe Peptides (salmon protein isolate) SalMe Collagen Peptides (collagen peptides sourced from salmon) SalMe Salmon Oil (Food-grade salmon oil) Pet Food Ingredients: Salmigo^®^ Protect L60 Salmigo^®^ Active (highly digestible salmon protein powder) Salmigo^®^ Salmon Oil Aquaculture Feed Ingredients: Salmon Oil for Aquafeed (omega-rich feed ingredient)
Hofseth BioCare [144] (Norway) Salmon byproducts (16,145 t/2024)	OmeGo^®^ (salmon oil) ProGo^®^ (protein hydrolysate) CalGo^®^ (calcium/collagen) PetGo™ (non-soluble protein)
Thai Union Ingredients [145] (Thailand) [145] Tuna waste (15,000 t/year) *	ThalaCol™ (Marine Collagen Peptides) UniQ™DHA (Omega-3 Oils) UniQ^®^COL (Protein Hydrolysates) UniQ^®^BONE (Tuna bone powder, source of calcium, phosphorus and collagen)
TripleNine Group [146] (Denmark) [146] Trimmings from various fisheries ** (~110,000 t/year, TripleNine Lota, Chile)	Marine ingredients for the aquaculture: SUPERPRIME fishmeal PRIME fishmeal STANDARD fishmeal
Valora Marine Ingredients [147] (Spain) Seafood processing byproducts ***	Functional ingredients for food, pharmaceutical, and cosmetic industries Natural seasonings & flavour enhancers Pet Food/animal ingredients Ingredients for agriculture
Bio-Marine [148] (Ireland) Blue whiting and other under-utilised species (~14,000 t/year raw marine processing, Monaghan, Ireland)	Nutritional and functional ingredients: ProAtlantic (hydrolysed protein isolate (~95%) for human nutrition) Proshore (protein concentrates (55–90%) for pet food and aquaculture nutrition) WhiteCal (products rich in calcium from fish bones) Omega-Blue and lipid powders (lipid/oil fractions for food/pet foods/aquaculture) ProGlas (Biofertilisers for agriculture)

* This value was estimated using the 1500 t/year of marine collagen from tuna skin output, a assuming a typical collagen yield recovery of 10%. ** The processed feedstook is partially coming from byproducts according to certified traceability information (e.g., MarinTrust). *** The Valora company operates within the Jealsa group processing ecosystem, where ≈20% of raw materials are directed to new valorisation processes, however the amount of processed raw materials is not available.

Besides the companies mentioned above, there are also several initiatives and projects focused on the utilisation of marine byproducts, particularly aimed at the production of sustainable, high-value food and feed ingredients from underutilised marine biomass that may result in sustainable solutions for the future, with some being outlined below.

The FOODIMAR Project [149] (*Sustainable climate-friendly quality food ingredients from marine side-streams*) is currently testing three pilot-scale approaches. The first pilot focuses on the utilisation of fisheries side-streams from Atlantic cod (*Gadus morhua*), Saithe (*Pollachius virens*), and Haddock (*Melanogrammus aeglefinus*), aiming at the recovery of collagen, gelatin, and glycosaminoglycans. The side-streams are mainly processed via silage, resulting in feed ingredients for human consumption in markets such as Africa. The second pilot evaluates the utilisation of jellyfish by-catch, targeting Moon jellyfish (*Aurelia aurita*) and Comb jellyfish (*Mnemiopsis leidyi*). This pilot is particularly relevant because jellyfish in Denmark are currently neither consumed nor utilised, resulting in an immature, exploratory, and non-regulated value chain. The objectives of this pilot include identifying both opportunities and obstacles, as well as the full characterization of jellyfish-derived ingredients. Potentially, this pilot may contribute to the creation of a new supply chain for the seafood sector. The third pilot includes both aquaculture side-streams and jellyfish by-catch, targeting Seabream (*Sparus aurata*) and jellyfish (*Aurelia aurita*, *Rhizostoma pulmo*, *Rhopilema nomadica*, and *Pelagia noctiluca*). This pilot pursues aims like those of the previous pilots, focusing on ingredient recovery and valorisation pathways.

Another relevant example of marine sustainability initiatives is MARMADE [150] (*Marine biomass valorization for food and feed innovation*), a Circular Bio-based Europe–funded project that utilises shrimp shells, blue crabs, and other crustaceans. The project aims to establish advanced biorefinery processes for the extraction of compounds such as peptides, prebiotics, lipids, and vitamins.

The BLUE BIOECONOMY PACT [151] is a Portuguese consortium that has embraced the challenge of reindustrializing national industries through the integration of blue biotechnology solutions into value chains. The initiative leverages the sustainable use of marine bioresources to increase added value through decarbonising innovation. Among its transversal initiatives, the pact includes overarching projects such as a digital platform dedicated to the valorisation of seafood byproducts, with an emphasis on their traceability, including the confirmation of their geographic origin.

Finally, the LIFE REFISH Project [152] (*Flexible biorefinery to valorise discards and byproducts of the European fish and seafood production*, 2022–2025) addresses the entire value chain, from determining optimal storage conditions to demo-scale biorefinery testing (300 kg byproducts. h^−1^), identifying potential commercial value, and preparing an industrial-scale process (4 t. h^−1^) for commercialisation.

Rather than viewing so-called “byproducts” as waste, this review reinforces the need to recognise them as potential co-products that, when processed appropriately, can generate significant added value and new revenue streams. Through cascading and multi-stage valorisation approaches, a single biomass source can yield multiple co-products, transforming food loss and waste from an environmental and economic burden into an opportunity for sustainable production [142]. Such strategies contribute to income diversification for producers without increasing primary production pressure and provide additional food and material resources to meet growing global demand. Importantly, cascading use of biomass can also reduce competition among end-use sectors, if byproducts are sufficiently characterised to enable their targeted and safe application—an aspect closely linked to food security, for which traceability of geographic origin is essential [153]. Given their high content of valuable components, seafood byproducts should therefore be prioritised for upcycling valorisation rather than simple recycling. As future challenges, no process can be considered complete or successful without incorporating techno-economic analysis and life cycle assessment (LCA) studies, which are essential for fully understanding the economic feasibility and environmental impacts of the developed processes [154].

## 5. Conclusions

The central question addressed in the present work is whether the geographic origin of marine byproducts can be reliably traced using their composition, particularly in the absence of reliable information from where they were farmed or captured. The answer is affirmative, but not in a direct or straightforward manner. The reliable tracing of geographic origin of seafood byproducts requires critical control and interpretation of all processing steps, as each one of them holds the potential to induce significant shifts to the compositional and structural features of the markers selected. When sufficient and well-characterised data are made available, these changes may be effectively modelled, hence enabling a prediction of a specific origin. However, this requires a careful balance between the complexity of information obtained from minimally processed materials—which may include confounding contributions from heterogeneous structures—and the cleaner, but potentially altered, information derived from purified extracts. A comprehensive approach must therefore integrate multiple layers of information to reconstruct the processing pathway, often as far back as to its original source.

The first essential step to trace the origin of a given seafood byproduct is the characterisation of the byproduct itself. Although mixed byproducts may be analysed, the contribution of each component should be considered individually. In this study, four key stages were identified as critical when aiming to trace the origin of a seafood byproduct: (1) overview of the byproduct, (2) compositional analysis, (3) direct analysis and/or extraction conditions, and (4) purification.

Step 1—Overview of the byproduct. This stage defines subsequent analytical choices. Water content is a critical initial parameter, as it determines byproduct stability and the need for stabilisation strategies [7]. Because byproducts consist of living or recently living tissues, time and moisture strongly influence compositional integrity [155]. High-moisture matrices deteriorate rapidly if not processed promptly, leading to loss of traceable features [32]. Scales and bones are generally more stable and display lower moisture levels, whereas skin exhibits higher and more variable water content depending on species and initial processing. Crustacean shells are initially low in moisture, but their recovered products, namely chitin, may absorb water during storage, requiring controlled handling conditions [156].

Step 2—Composition. Overall composition can be assessed using complementary analytical techniques. TGA provides an overview of water content, organic decomposition, and inorganic residue, offering a first compositional fingerprint [109]. FTIR enables identification of functional groups that can be correlated to the major families of components—proteins, lipids, carbohydrates, and minerals—and provides structural information related to key compounds such as collagen and HAp [17,96]. EDX can reflect organic–inorganic interactions, for example, through deviations in Ca/P ratios caused by collagen-entrapped Hap [96]. Collagen is most commonly extracted from skin due to higher yields and milder extraction requirements, whereas gelatin can be recovered from skin, bones, and scales under harsher conditions. Chitin and the derivatised chitosan can be retrieved from crustacean shells, while minerals are present in all byproducts, with bones and scales serving as phosphate sources and shells as carbonate sources. Lipids are particularly abundant in roe and in the skin of oil-rich species. While oil recovery is often a primary objective in conventional processing, advanced valorisation strategies aim to control lipid interference to enable the extraction of higher-value compounds such as proteins [55,121].

Step 3—Direct analysis and extraction conditions. Whenever feasible, direct analysis of minimally treated samples is preferred to trace the origin of seafood byproducts, as it preserves intrinsic features. However, most analytical techniques require some degree of purity, making extraction unavoidable. Extraction conditions must therefore be carefully selected, as they influence both yield and structural integrity. Collagen extraction requires deproteinization and demineralization, whereas chitin/chitosan recovery involves additional depigmentation and deacetylation steps [12,20,43]. Lipid-rich matrices pose particular challenges, as lipids interfere with protein extraction and characterisation [55]. Chemical and enzymatic treatments not only affect yield but also modify molecular structure, which must be considered when interpreting markers for traceability. Notably, peptide composition often remains informative even when higher-order structures are altered [65].

Step 4—Purification. Purification further alters compositional balance and impacts analytical outputs. For example, TGA profiles are strongly influenced by organic–inorganic ratios, with characteristic mass losses associated with water evaporation, organic decomposition, and mineral stability at higher temperatures [109]. These patterns can serve as fingerprints that help tracing origin when the composition and processing history are known. It may be hypothesised that with sufficient data, modelling approaches may eventually allow the prediction of byproduct behaviour under non-purified conditions, approximating the original material state.

Among compositional markers, collagen emerges as a key indicator due to its abundance in skin, bones, and scales. Parameters such as nitrogen content, imino acid composition (proline and hydroxyproline), crosslinking density, and thermal stability provide valuable information when aiming to trace the origin of seafood byproducts when extraction-induced shifts are carefully controlled. Collagen denaturation temperature, measurable by viscosity or thermal techniques, reflects habitat conditions. Mineral-based markers from bones and scales further complement protein-based approaches. Variations in hydroxyapatite composition—such as Ca/P ratio, carbonate substitution, trace element content, and thermal behaviour—generate inorganic fingerprints linked to species, habitat, and diet. Crustacean shells, characterized by chitin and calcium carbonate rather than hydroxyapatite, retain mineral features detectable by thermal and spectroscopic methods, despite extensive chemical processing. Fish roe represents a nutritionally rich byproduct and a major source of biologically active vitamin B12; while morphological identification is unreliable, proteomic tools such as MALDI-TOF MS provide rapid and cost-effective species discrimination to reinforce the information that can be retrieved from the lipid profile.

Overall, no single compositional marker is sufficient to ensure the reliable tracing of the geographic origin of seafood byproducts. The integration of complementary organic and inorganic markers with well-documented processing conditions and multi-analytical strategies will most likely be required. A thorough understanding of composition, combined with targeted industrial and commercial strategies, is essential to fully harness the value of marine byproducts framed within circular bioeconomy models.

## Figures and Tables

**Table 1:** Analytical techniques used for composition characterization.

Compound	Analytical Characterization
Protein hydrolysate	(**Skin**) UPLC-MS [113]; HPLC, UV-Vis, FTIR, SEM, DSC, XRD, ELS [8] (**Roe**) GC-FID [16]; HPLC [74] (**Mix**) UPLC [1]; HPLC, ATR-FTIR, SEC [7]
Collagen	(**Skin**) LC-MS/MS [3]; SDS-PAGE, FTIR [63]; SDS–PAGE, FTIR [61] SDS-PAGE, Ho, DLS, LC-MS/MS [48]; (**Bones**) SDS-PAGE, UV-Vis [69]; SDS-PAGE, FTIR, UV-Vis [56] (**Scales**) SEM, FTIR, UV-Vis, XRD, TGA, SDS–PAGE [21]
Gelatin	(**Skin**) UPLC, SEM, FTIR [24]; HPLC, LC-MS, FTIR [65] (**Scales**) FTIR, UPLC [4]; TGA, FTIR, DLS, ELS, SEM [17]; SDS–PAGE, GPC, Viscoelastic studies [64]; FTIR, XRD, TEM-EDS, BET, TGA [46]; High-Speed Amino Acid Analyzer, UPLC, FTIR, SEM, ELS,^1^H NMR, TG-DSC [41];
Minerals	(**Skin**) ICP-MS [3] (**Scales**) ICP-OES [17]
Hydroxyapatite	(**Bones**) TEM, XDR, FTIR, ESR [110]; XRD, EDS, BET, SEM, HR-TEM, ELS [104]; FTIR, XRD, SEM, FTIR [99]; UV-Vis, FTIR, TEM, XRD [94]; SEM, TEM, EDS, XRD [90]; XRD, FTIR, TEM [88]; WDXRF, FTIR, XRD, SEM-EDX, BET [71]; FTIR, XRD, SEM [69]; XRD, FTIR, SEM, TEM, ICP-MS [67]; SEM, FTIR, XRD, AFM [62]; TG-DTA, FTIR, XRD, SEM-EDX, XRF [10] (**Scales**) XRD, FTIR, TGA, SEM, EDX [96], SEM [73]; XRD, EDS [45]; (**Shells**) SEM [12]; DLS, FTIR, XRD, FESEM, EDAX, HRTEM, TG, DTA, AFM, ELS [109]
Calcium	(**Bones**) XRD, SEM, EDX, FTIR [11] (**Shells**) FE-SEM, EDX, FTIR [5]
Chitosan	(**Shells**) FTIR-ATR, 13C CPMAS NMR, SEM, TGA, DTG, DTA [12]; FTIR, XRD, TGA, EDX [80]; FTIR [20]; FTIR, XRD, SEM, BET [43]; SEM, EDX, BET, FTIR, XRD, TGA [42]
Vitamin B12	(**Roe**) LC–MS/MS [75]
Phospholipids	(**Roe**) ^31^P NMR [16]

Spectroscopy-based methods: 13C CPMAS NMR—Carbon-13 Cross-Polarization Magic-Angle Spinning Nuclear Magnetic Resonance; ^1^H NMR—Proton Nuclear Magnetic Resonance; ^31^P NMR—Phosphorus-31 Nuclear Magnetic Resonance; ESR—Electron Spin Resonance; FTIR-ATR—Fourier Transform Infrared Spectroscopy (Attenuated Total Reflectance); UV-Vis—Ultraviolet–Visible Spectroscopy. Microscopy-based methods: AFM—Atomic Force Microscopy; FESEM—Field Emission Scanning Electron Microscopy; HRTEM—High-Resolution Transmission Electron Microscopy; SEM—Scanning Electron Microscopy. Chromatography-based methods: LC-MS/MS—Liquid Chromatography–Tandem Mass Spectrometry; SEC—Size Exclusion Chromatography; UPLC-MS—Ultra-Performance Liquid Chromatography–Mass Spectrometry. Molecular characterization: SDS-PAGE—Sodium Dodecyl Sulfate–Polyacrylamide Gel Electrophoresis. Elemental analysis: EDX—Energy-Dispersive X-ray Spectroscopy; ICP-MS—Inductively Coupled Plasma–Mass Spectrometry; ICP-OES—Inductively Coupled Plasma–Optical Emission Spectroscopy; WDXRF—Wavelength-Dispersive X-ray Fluorescence; XRF—X-ray Fluorescence Spectroscopy. Thermal analysis: DSC—Differential Scanning Calorimetry; DTG—Derivative Thermogravimetry; DTA—Differential Thermal Analysis; TGA—Thermogravimetric Analysis; TG-DTA—Thermogravimetric–Differential Thermal Analysis. Interfacial properties: BET—Brunauer–Emmett–Teller adsorption–desorption; DLS—Dynamic Light Scattering; ELS—Electrophoretic Light Scattering (zeta potential); Ho—Surface Hydrophobicity. Other: XRD—X-ray Diffraction.

**Table 2:** Proximate composition and extraction yields of skin, bone, scale, and cartilage-derived collagen and gelatin from marine species.

	Cold-Water Marine Fish Species									Temperate and Tropical Marine Species									
	Cod (*Gadus morhua*)			Atlantic salmon (*Salmo salar*)						Bigeye snapper (*Priacanthus tayenus*)				Blacktip shark (*Carcharhinus limbatus*)			Brownbanded bamboo shark (*Chiloscyllium punctatum)*		
	Skin ASC [128]	Skin SG [65]	Skin PSG [65]	Skin [121]	Skin L/M/H ASG [129]	Bones [121]	Scales ASC [21]	Scales PSC [21]		Skin [130]	Skin ASC [130]	Bone [130]	Bone ASC [130]	Cart. [127]	Cart. ASC [127]	Cart. PSC [127]	Cart. [127]	Cart. ASC [127]	Cart. PSC [127]
Moisture	9.7	4.8	6.8	52.7	6.6/4.0/3.9	64.3				64.08	7.06	62.27	11.57	70.29	7.85	8.07	66.84	6.54	6.62
Protein (%) C/non-C (dry weight)	26.4 21.5/4.9 (71.2/16.3)	94.4	92.0	20.0	87/94/99	14.1				32.0	94.0	13.3	84.2	14.85	90.47	90.18	14.01	90.70	90.91
Ash (dw)	(12)	0.9	1.1	1.0	0.8/0.6/0.6	6.4				3.23	0.68	14.40	0.88	(12.09)	(0.76)	(0.70)	(15.79)	(0.81)	(0.74)
Fat (dw)	(1)			26.3		15.2				0.98	0.33	8.77	0.48	(0.21)	(0.38)	(0.34)	(0.23)	(0.42)	(0.46)
Yield (g/100 g sample)							0.87	2.03			10.94		1.59		1.04	10.3		1.27	9.59
Collagen/gelatin extractability (g/100 g protein)	85	51	58																
Mw (kDa)		159	97		65/95/173														
Hyp (mg/g sample)	14.6	n.d.	n.d.		67.5/63.2/81.4		35.1	58.4		19.5	58.5	5.71	42.5	13.4	85.8	88.9	11.4	103.7	104.5
(mg/g protein)	55.3	n.d.	n.d.		58.7/59.4/80.6					60.9	62.2	42.9	50.5	90.5	94.8	98.6	81.2	114.3	114.9

Abbreviations: ASC, acid-soluble collagen; PSC, pepsin-soluble collagen; SG, soluble gelatin (40 °C); PSG, pancreatin-soluble gelatin; ASG, acid-soluble gelatin; Cart., cartilage; Mw, molecular weight; L/M/H, low-, medium-, and high-molecular-weight gelatin. C, Collagen; non-C, Non-collagenous proteins; Protein content calculated using N × 6.25 or Hyp × 14.7, or N × 5.5.

**Table 3:** Amino acid composition and thermal properties of collagen, gelatin, and collagen-derived protein hydrolysates from skin, bone, and cartilage (residues per 1000 residues).

	Cold-Water Marine Fish Species										Temperate and Tropical Marine Species					
Amino Acids	Atlantic cod (*Gadus morhua*)					Salmon (*Salmo salar*)			Salmon (*Oncorhynchus* *nerka*)		Bigeye snapper (*Priacanthus* *tayenus*)		BTS (*Carcharhinus* *limbatus*)		BBS (*Chiloscyllium* *punctatum*)	
	Skin ASC [131]	Skin ASC (Control) [132]	Skin ASC (Starved) [132]	Skin SG [65]	Skin PSG [65]	Skin ASG-L [129]	Skin ASG-M [129]	Skin ASG-H [129]	Skin PH [55]		Skin ASC [130]	Bone ASC [130]	Cart. ASC [127]	Cart. PSC [127]	Cart. ASC [127]	Cart. PSC [127]
Gly	342	347	333	342	298	423	402	457	359		286	361	317	316	317	317
Pro	103	103	106	103	88	98	98	125	93		116	95	109	106	105	110
Ala	107	107	107	107	127	114	115	92	80		136	129	104	118	119	104
Glu	80	74	72	80	83	93	90	89	65		78	74	78	77	77	77
Arg	54	57	57	54	70	52	57	55	52		60	46	51	54	54	51
Asp/Asn	53	53	51	53	65	55	62	41	55		51	47	42	43	42	43
Ser	59	69	67	59	70	49	49	52	50		36	34	41	31	30	41
Leu	22	20	21	22	35	17	18	11	32		24	25	24	26	25	25
Thr	23	25	24	23	29	19	20	17	32		29	25	23	22	21	24
Phe	12	12	12	12	18	12	13	11	14		15	12	14	13	14	13
Lys	29	30	27	29	38	34	34	27	43		31	25	28	26	27	27
Val	19	15	15	19	28	9	13	5	24		22	17	25	26	26	25
Met	15	16	18	15	15	14	15	11	15		12	8	13	14	14	12
Ile	12	9	9	12	18	8	9	5	14		5	5	18	20	19	19
Tyr	4	5	4	4	8	n.d.	n.d.	n.d.	8		4	2	3	3	3	3
His	8	9	9	8	13	4	6	2	14		10	6	7	8	8	7
Hyp	51	52	54	n.d.	n.d.	75	74	84	44		77	68	94	91	91	94
Hyl	7	n.d.	n.d.	n.d.	n.d.	n.d.	n.d.	n.d.	5		10	20	7	8	7	7
Cys	n.d.	n.d.	n.d.	n.d.	n.d.	n.d.	n.d.	n.d.	n.d.		n.d.	n.d.	1	1	1	1
P_DH_	33.1	33.8	34.0	n.d.	n.d.	53.2	53.2	53.2	32.1		39.9	41.7	46.3	46.2	46.4	46.1
Ia	154	155	160	n.d.	n.d.	174	172	208	137		193	163	203	197	196	204
T_D_	15.0 ^1^	12.2 ^2^	12.7 ^2^								31.0	35.5	36.3	34.6	36.7	36.0
T_gel_				7.4 ^3^	−0.3 ^3^	3.3 ^2^/2.8 ^3^	6.9 ^2^/7.0 ^3^	10.3 ^2^/10.3 ^3^								
T_M,gel_				16.4 ^3^	10.1 ^3^											

^1^ determined using the half viscosity measurement [131]. ^2^ determined using differential scanning calorimetry [127]. ^3^ determined using the dynamic viscoelastic profile [65]. Abbreviations: BTS, blacktip shark (*Carcharhinus limbatus*); BBS, brownbanded bamboo shark (*Chiloscyllium punctatum*); ASC, acid-soluble collagen; PSC, pepsin-soluble collagen; SG, soluble gelatin (40 °C); PSG, pancreatin-soluble gelatin; ASG, acid-soluble gelatin; PH, collagen-derived protein hydrolysates; P_DH_, proline degree of hydroxylation; Cart., cartilage; Mw, molecular weight; L/M/H, low-, medium-, and high-molecular-weight gelatin; Ia, imino acids (Pro + Hyp); T_D_, collagen denaturation temperature; T_gel_, gelatin gelling temperature; T_M,gel_, gelatin melting temperature; n.d., not determined.

**Table 4:** Collagen types identified in raw fish skin and corresponding collagen-derived protein hydrolysates using emPAI analysis [3].

	Atlantic Cod (*Gadus morhua*)		Atlantic salmon (*Salmo salar*)		White Fish (*Platichthys flesus*)	
**Type of collagen**	Skin	PH	Skin	PH	Skin	PH
**Type I**, [α1(I)]_2_ + α2 (I)						
Collagen α1 (I)	0.143		0.218	0.081	0.219	
Collagen α2 (I)	0.182	0.822	0.513	0.374	0.061	0.066
**Ratio (α1/α2) (I)**	0.800	-	0.400	0.200	3.600	-
**Type II**, [(α-1(II)]_3_	0.035		0.030			
**Type III**, [(α-1(III)]_3_					0.039	0.059
**Type V** Collagen α-1 (V)	0.023		0.049			
**Type XII** Collagen α-1 (XII)			0.006		0.014	
**Type XV** Collagen α-1 (XV)		0.056		0.035		0.035

Collagen types include fibrillar (I, II, III), FACIT (XII), and multiplexin collagens (XIII, XV). Abbreviations: PH, collagen derived Protein Hydrolysates.

**Table 5:** Estimated elements (%) content in seafood skin, scales, bones, and shells.

		Skin		Scales				Bones						Shell
		Atlantic cod (*Gadus morhua*)	Atlantic salmon (*Salmo salar*)	Mullet (*Mugil cephalus*)		Seabass (*Dicentrarchus* *labrax*)	Seabass (*Lates* *calcarifer*)	Baltic cod (*Gadus morhua* *callarias*)		Atlantic salmon (*Salmo salar*)		Gilthead seabream (*Sparus aurata)*	Yellowfin tuna (*Thunnus* *albacares*)	Shrimp (*Penaeus monodon*)
		[3]	[3]	[17]		[17]	[96]	[116]		[116]		[88]	[5]	[5]
Moisture (%, db)				Raw	SG	Raw	Raw	Raw	P	Raw	P	P	P	
				10	11	11	10	78.42	5.97	65.45	5.34		0.98	3.45
Protein (%,db)				(52)		(44)		15.27	14.20	18.02	10.78		30.28	37.78
Ash (%, db)				(35)		(40)		5.91	73.28	4.09	70.15		62.63	36.58
Lipid (%,db)				(0.4)		(0.9)		0.76	0.25	12.3	0.12		7.70	2.67
Major (bulk) elements (g/kg)	C			231	392	213	389				221.9	162.9	221.9	545.4
	H			44	66	44								
	N			83	150	71								
	S			4	50	4						-		
	O						526				368.8	495.5	368.8	345.4
Macrominerals (g/kg)	P	10	5		4		40		134.0		125.0	120.0	138.5	12.6
	Ca	2.0	3.9		5		38		277.9		249.2	190.0	245.2	75.8
	Na								12.3		10.0	6.58	19.0	15.3
	Mg	0.75	0.5						6.6		4.6	20.6	6.4	3.7
	K	2.4	3.0						0.30		0.27		0.2	1.4
Microminerals (mg/kg)														
Trace elements (µg/kg)	Fe	4500	12,900						24,000		11,000		20	40
	Cu	600	600						62,000		3900			
	Zn	50	25						50,000		57,000			
Ca/P ratio				2.17		2.16	0.74		1.99		2.07	1.57	1.77	6.01

Abbreviations: SG, soluble gelatin; P, preparate.

**Table 6:** Moisture, protein, Vitamin B12, and lipid contents, and fatty acid distribution (%) in Roe.

	Hoki (*Macruronis* *novaezelandiae*)		Gemfish (*Rexea* *solandri)*	Blue Mackerel (*Scomber* *australasicus*)	Kina (Sea Urchin) (*Evechinus chloroticus*)	Red Cod (*Pseudophycis bacchus*)	Chum Salmon “Ikura” (*Oncorhynchus* *keta*)	Alaska Pollock “Tarako” (*Gadus* *chalcogrammus)*	Flying Fish “Tobiko” (*Hirundichthys oxycephalus)*	Pacif Herring “Kazunoko” (*Clupea* *pallasii*)
	[16]	[138]	[16]	[138]	[138]	[138]	[137]	[137]	[137]	[137]
Moisture (%)	68.8 ± 0.38		63.2 ± 0.70							
Protein (%)	17.9 ± 0.72		23.8 ± 0.74							
Ash (%)	1.3 ± 0.12		1.3 ± 0.83							
Carb. (%)	1.9 ± 0.83		4.2 ± 0.64							
B12 (µg/100 g)							14.2 ± 6.4	4.7 ± 0.3	3.9 ± 0.2	
PL (%)							5.51 ± 0.12	2.28 ± 0.23	2.39 ± 0.19	2.14 ± 0.26
PC (%, of PL)	28.7 ± 6.2		34.6 ± 6.5				77.9 ± 0.1	68.5 ± 0.5	76.6 ± 0.9	65.2 ± 5.8
Lipids (%)	10.1 ± 0.65	12.3	7.6 ± 0.03	7.3	5.3	9.6	14.5 ± 0.7	3.7 ± 0.4	3.2 ± 0.2	3.0 ± 0.3
Fatty acid (%)										
C14:0	3.0 ± 0.09	2.9	1.4 ± 0.09	2.9	19.8	1.8	4.6 ± 0.1	2.4 ± 0.1	1.4 ± 0.0	2.3 ± 0.2
C16:0	14.8 ± 0.3	15.0	12.5 ± 0.30	18.2	14.5	14.4	11.6 ± 0.2	21.8 ± 0.5	25.5 ± 0.2	26.3 ± 0.5
C18:0	2.4 ± 0.26	2.1	4.4 ± 0.26	4.2	2.3	1.7	4.6 ± 0.1	2.4 ± 0.0	9.8 ± 0.1	2.6 ± 0.4
C20:0	0.6 ± 0.12	Tr.	0.3 ± 0.08	0.1	0.4	tr.				
C22:0	1.4 ± 0.30		1.2 ± 0.09							
C22:0	0.3 ± 0.03		0.4 ± 0.04							
Others							0.9 ± 0.2	0.4 ± 0.0	2.9 ± 0.0	0.8 ± 0.2
**SFA (total)**	23.7 ± 1.35		20.9 ± 0.62				21.6 ± 0.2	26.9 ± 0.6	39.6 ± 0.3	32.0 ± 1.3
C14:1	4.8 ± 0.2	0.3		0.2	1.3	0.2				
C16:1	0.6 ± 0.06	7.9	0.3 ± 0.01	5.1	6.4	5.9				
C16:1 *n*-9							0.4 ± 0.0	0.5 ± 0.0	0.3 ± 0.0	0.6 ± 0.0
C16:1 *n*-7							5.6 ± 0.1	3.3 ± 0.0	1.9 ± 0.0	4.8 ± 0.3
C18:1 *n*-12							1.9 ± 0.0	0.9 ± 0.0		0.5 ± 0.0
C18:1 *n*-11	3.9 ± 0.25		4.3 ± 0.28							
C18:1 *n*-9	23.7 ± 1.94	28.8	29.4 ± 0.61	21.6	9.9	29.3	17.9 ± 0.2	9.3 ± 0.1	8.9 ± 0.1	12.1 ± 0.1
C18:1 *n*-7							3.1 ± 0.1	5.4 ± 0.1	2.4 ± 0.1	5.2 ± 1.0
C18:1 *n*-5							0.6 ± 0.0	0.6 ± 0.0		0.5 ± 0.0
C20:1	4.7 ± 0.58	5.3	3.6 ± 0.21	1.8	15.1	8.4	2.4 ± 0.0	3.1 ± 0.0	0.5 ± 0.0	0.7 ± 0.0
C22:1	0.4 ± 0.05	1.9	0.3 ± 0.004	2.1	9.5	3.2				
C22:1 *n*-9	0.3 ± 0.10		0.3 ± 0.02							
**MUFA (total)**	40.2 ± 1.01	58.6	41.5 ± 4.40	69.3	48.0	76.6	33.1 ± 0.4	25.0 ± 0.3	14.4 ± 0.1	25.0 ± 0.9
C18:2 *n*-6	1.2 ± 0.03	1.5	1.1 ± 0.08	2.0	1.6	1.4	1.0 ± 0.0	1.0 ± 0.0	1.1 ± 0.0	0.7 ± 0.1
C18:3 *n*-3	0.5 ± 0.03		0.6 ± 0.05				0.7 ± 0.0	0.4 ± 0.0	0.7 ± 0.0	0.4 ± 0.0
C18:4 *n*-3							0.8 ± 0.0	0.8 ± 0.0	0.4 ± 0.0	0.4 ± 0.0
C20:2	0.3 ± 0.02		0.2 ± 0.03							
C20:3 *n*-3	0.9 ± 0.15		1.7 ± 0.04							
C20:4 *n*-6							1.0 ± 0.0	1.3 ± 0.0	3.0 ± 0.1	1.1 ± 0.1
C20:4 *n*-3							2.1 ± 0.0	0.6 ± 0.0	0.5 ± 0.0	0.4 ± 0.0
C20:5 *n*-3 (EPA)	8.1 ± 0.90	8.2	5.7 ± 0.41	10.1	3.7	6.6	13.6 ± 0.1	18.8 ± 0.3	7.0 ± 0.1	15.0 ± 0.6
C22:4		0.9		0.8	0.2	1.2				
C22:4 *n*-6							0.2 ± 0.0			0.2 ± 0.0
C22:5 *n*-3	1.9 ± 0.30	1.5	4.3 ± 0.20	1.8		1.5	5.6 ± 0.0	1.3 ± 0.0	2.8 ± 0.0	1.3 ± 0.1
C22:6 *n*-3 (DHA)	18.6 ± 1.63	19.2	21.4 ± 0.59	22.6	0.2	19.8	17.4 ± 0.2	22.2 ± 0.4	27.9 ± 0.3	22.6 ± 1.0
Others							2.0 ± 0.2	1.5 ± 0.1	2.2 ± 0.0	0.6 ± 0.3
**PUFA (total)**	31.8 ± 1.31		35.1 ± 1.12				44.6 ± 0.5	47.5 ± 0.9	45.50 ± 0.4	42.7 ± 0.3
EPA+DHA	26.7	27.4	27.1	32.7	3.9	26.4	31.0	41.0	39.3	40.1
*n*-3	30.0 ± 0.91	27.4	33.8 ± 0.91	32.7	3.9	26.4	40.2	44.1	39.3	40.1
*n*-6	1.36 ± 0.03		1.84 ± 0.03				2.2	2.3	4.1	2.0
*n*-6/*n*-3	0.045		0.054				0.055	0.052	0.104	0.050

Abbreviations: Carb., Carbohydrates; PL, Phospholipids; PC, Phosphatidylcholine; EPA, eicosapentaenoic acid; DHA, Docosahexaenoic acid; SFA, saturated fatty acids; MUFA, monounsaturated fatty acids; PUFA, polyunsaturated fatty acids; tr, traces.

## Data Availability

No new data were created or analyzed in this study. Data sharing is not applicable to this article.

## Supplementary Materials

The following supporting information can be downloaded at: https://www.mdpi.com/article/10.3390/foods15061073/s1. Keyword information for Prisma analysis. PRISMA 2020 Main Checklist. Reference [157] is cited in the Supplementary Materials.
