# Supplementary material for: Criteria for the Characterization of Seafood Byproducts to Allow Tracing Their Geographic Origin
Source: Foods. 2026 Mar 18;15(6):1073. doi: 10.3390/foods15061073 (PMC13024785; doi:10.3390/foods15061073)
Supplement: Supplementary file 1 [file foods-15-01073-s001.zip › foods-4119672-supplementary.pdf]

PRISMA 2020 Main Checklist

Topic	No.	Item	Location where item is reported
**TITLE**			
**Title**	1	Identify the report as a systematic review.	line 1
**ABSTRACT**			
**Abstract**	2	See the PRISMA 2020 for Abstracts checklist	
**INTRODUCTION**			
**Rationale**	3	Describe the rationale for the review in the context of existing knowledge.	Section 3.2
**Objectives**	4	Provide an explicit statement of the objective(s) or question(s) the review addresses.	line 178-179
**METHODS**			
**Eligibility criteria**	5	Specify the inclusion and exclusion criteria for the review and how studies were grouped for the syntheses.	Section 2.2
**Information sources**	6	Specify all databases, registers, websites, organisations, reference lists and other sources searched or consulted to identify studies. Specify the date when each source was last searched or consulted.	section 2.1
**Search strategy**	7	Present the full search strategies for all databases, registers and websites, including any filters and limits used.	Sections 2.1 and 2.2
**Selection process**	8	Specify the methods used to decide whether a study met the inclusion criteria of the review, including how many reviewers screened each record and each report retrieved, whether they worked independently, and if applicable, details of automation tools used in the process.	section 2.2
**Data collection process**	9	Specify the methods used to collect data from reports, including how many reviewers collected data from each report, whether they worked independently, any processes for obtaining or confirming data from study investigators, and if applicable, details of automation tools used in the process.	section 2.2

Topic	No.	Item	Location where item is reported
**Data items**	10a	List and define all outcomes for which data were sought. Specify whether all results that were compatible with each outcome domain in each study were sought (e.g. for all measures, time points, analyses), and if not, the methods used to decide which results to collect.	section 2.1 and 2.2
	10b	List and define all other variables for which data were sought (e.g. participant and intervention characteristics, funding sources). Describe any assumptions made about any missing or unclear information.	not define
**Study risk of bias assessment**	11	Specify the methods used to assess risk of bias in the included studies, including details of the tool(s) used, how many reviewers assessed each study and whether they worked independently, and if applicable, details of automation tools used in the process.	not define
**Effect measures**	12	Specify for each outcome the effect measure(s) (e.g. risk ratio, mean difference) used in the synthesis or presentation of results.	not define
**Synthesis methods**	13a	Describe the processes used to decide which studies were eligible for each synthesis (e.g. tabulating the study intervention characteristics and comparing against the planned groups for each synthesis (item 5)).	not define
	13b	Describe any methods required to prepare the data for presentation or synthesis, such as handling of missing summary statistics, or data conversions.	section 2.3
	13c	Describe any methods used to tabulate or visually display results of individual studies and syntheses.	section 2.3
	13d	Describe any methods used to synthesize results and provide a rationale for the choice(s). If meta-analysis was performed, describe the model(s), method(s) to identify the presence and extent of statistical heterogeneity, and software package(s) used.	section 3.2
	13e	Describe any methods used to explore possible causes of heterogeneity among study results (e.g. subgroup analysis, meta-regression).	not provided
	13f	Describe any sensitivity analyses conducted to assess robustness of the synthesized results.	not provided
**Reporting bias assessment**	14	Describe any methods used to assess risk of bias due to missing results in a synthesis (arising from reporting biases).	not provided
**Certainty assessment**	15	Describe any methods used to assess certainty (or confidence) in the body of evidence for an outcome.	not provided

Topic	No.	Item	Location where item is reported
**RESULTS**			
**Study selection**	16a	Describe the results of the search and selection process, from the number of records identified in the search to the number of studies included in the review, ideally using a flow diagram.	Figure 1
	16b	Cite studies that might appear to meet the inclusion criteria, but which were excluded, and explain why they were excluded.	section 2.2
**Study characteristics**	17	Cite each included study and present its characteristics.	line 214
**Risk of bias in studies**	18	Present assessments of risk of bias for each included study.	not included
**Results of individual studies**	19	For all outcomes, present, for each study: (a) summary statistics for each group (where appropriate) and (b) an effect estimate and its precision (e.g. confidence/credible interval), ideally using structured tables or plots.	Figures 4-6 and 7-9
**Results of syntheses**	20a	For each synthesis, briefly summarise the characteristics and risk of bias among contributing studies.	not included
	20b	Present results of all statistical syntheses conducted. If meta-analysis was done, present for each the summary estimate and its precision (e.g. confidence/credible interval) and measures of statistical heterogeneity. If comparing groups, describe the direction of the effect.	not included
	20c	Present results of all investigations of possible causes of heterogeneity among study results.	not included
	20d	Present results of all sensitivity analyses conducted to assess the robustness of the synthesized results.	not included
**Reporting biases**	21	Present assessments of risk of bias due to missing results (arising from reporting biases) for each synthesis assessed.	not included
**Certainty of evidence**	22	Present assessments of certainty (or confidence) in the body of evidence for each outcome assessed.	not included
**DISCUSSION**			
**Discussion**	23a	Provide a general interpretation of the results in the context of other evidence.	Section 3.2
	23b	Discuss any limitations of the evidence included in the review.	Section 3.2

Topic	No.	Item	Location where item is reported
	23c	Discuss any limitations of the review processes used.	Section 3.2
	23d	Discuss implications of the results for practice, policy, and future research.	section 3.2
**OTHER INFORMATION**			
**Registration and protocol**	24a	Provide registration information for the review, including register name and registration number, or state that the review was not registered.	Not registered
	24b	Indicate where the review protocol can be accessed, or state that a protocol was not prepared.	Figure 1
	24c	Describe and explain any amendments to information provided at registration or in the protocol.	Figure 1
**Support**	25	Describe sources of financial or non-financial support for the review, and the role of the funders or sponsors in the review.	line 985-992
**Competing interests**	26	Declare any competing interests of review authors.	not reported
**Availability of data, code and other materials**	27	Report which of the following are publicly available and where they can be found: template data collection forms; data extracted from included studies; data used for all analyses; analytic code; any other materials used in the review.	not included

PRIMSA Abstract Checklist

Topic	No.	Item	Reported?
**TITLE**			
**Title**	1	Identify the report as a systematic review.	yes
**BACKGROUND**			
**Objectives**	2	Provide an explicit statement of the main objective(s) or question(s) the review addresses.	Yes
**METHODS**			
**Eligibility criteria**	3	Specify the inclusion and exclusion criteria for the review.	Yes
**Information sources**	4	Specify the information sources (e.g. databases, registers) used to identify studies and the date when each was last searched.	Yes
**Risk of bias**	5	Specify the methods used to assess risk of bias in the included studies.	No
**Synthesis of results**	6	Specify the methods used to present and synthesize results.	Yes
**RESULTS**			
**Included studies**	7	Give the total number of included studies and participants and summarise relevant characteristics of studies.	Yes
**Synthesis of results**	8	Present results for main outcomes, preferably indicating the number of included studies and participants for each. If meta-analysis was done, report the summary estimate and confidence/credible interval. If comparing groups, indicate the direction of the effect (i.e. which group is favoured).	Yes
**DISCUSSION**			
**Limitations of evidence**	9	Provide a brief summary of the limitations of the evidence included in the review (e.g. study risk of bias, inconsistency and imprecision).	No
**Interpretation**	10	Provide a general interpretation of the results and important implications.	Yes
**OTHER**			
**Funding**	11	Specify the primary source of funding for the review.	Yes
**Registration**	12	Provide the register name and registration number.	No

From: Page MJ, McKenzie JE, Bossuyt PM, Boutron I, Hoffmann TC, Mulrow CD, et al. The PRISMA 2020 statement: an updated guideline for reporting systematic reviews. MetaArXiv. 2020, September 14. DOI: 10.31222/osf.io/v7gm2. For more information, visit: www.prisma-statement.org

Keyword information for Prisma analysis

The search strategy applied in this systematic review had into consideration the following research question: Can we define strategies to trace the origin of marine byproducts with potential for valorization? In order to provide a response, three main concepts were selected for keywords definition: Concept 1: "Trace the origen"; Concept 2: "Marine byproducts" and Concept 3: "Valorization". The search was performed assuming keywords based on "Trace the origen" and "Marine byproducts" or "Marine byproducts" and "Valorization".

The methodology applied was the Preferred Reporting Items for Systematic Reviews and Meta-Analyses (PRISMA) performing a search for publications in the SCOPUS, PubMed and Web of Science databases, using the following keywords: Concept C1: ("Authentic* of origin" or "Biochemi* Tools*"or "Biochemi* analis*" or Biomarkers or "Biotechnolog* Tools" or "Category of fishing gear" or "Certificate of origin" or "Combat fraud" or "DNA tools" or "DNA analys*" or "DNA barcoding" or "Element fingerprinting" or "Element* analys*" or "Fatty acid analys*" or "Feeding regimes" or Fingerprint or "Fisheries control" or "Food safety regulations" or "Food traceability" or "Fraudulent mislabelling" or "Geochemi* Tools" or "Geochemi* analys*" or "Geographic* Traceability" or "Harvesting location" or lipidomic or "Metabolomic tools" or "Metabolomic analys*" or Microarrays or "Molecular tools" or "Molecular analis*" or "Origin certification" or "Production location" or "Production method" or "Provenance" or "Quality requirements" or "safety requirements" or "species mislabeling" or "Stable isotope analysis" or "Sustainably manag* Fisheries" or "Trace* Orig*n" or "Trace* harvesting" or "Trace* processing/geographic orig*n" or "Trace* geographic orig*n" or "Unstable isotope analysis").

Concept C2: "(Aquaculture or mariculture or "Aquatic organism" or "Aquatic specie" or bioresources or "fish production" or "stock management" or "Fresh seafood" or Fish* or ecosystem or "Supply chain" or downstream or wastewater or biomass or "animal food" or organism) and ((Crustacean and head or shell or pleopods or tail or cephalothorax or hepatopancreas or intestine or roe or "male gonads" or liver or exoskeleton or carapace or waste or diadromous) or (Fish and co-products or eggs or "processing byproducts") or (Fish and head or brain or viscera or frame or skin or tail or fins or scale or mince or bones or thorns or blood or trimming or liver or stomach or fishbone or Intestine or offal or "skeletal frames" or gills or guts or "Minced side streams" or waste) or "Less savory parts" or (Marine and byproduct or coproduct or waste or "animal fat" or "processing byproducts" or "industry waste") or (seafood and byproduct or coproduct or waste or "processing byproducts" or "industry waste") or (Mollus* and shell or head or skin or viscera or tail* or ink or "soft tissues"or mantle or gill or liver or "digestive gland" or kidney or skin or stomach or intestine or "pyloric caeca" or waste) or Mucus or Non-edible or uneatable or "bioprocessing leftovers" or "biomass" or "biomass waste" or "biomass wastes" "fish skin" or "fish skin gelatin" or "fish waste" or "fishbone" or "waste biomass" or "waste fish oil").

Concept C3:("acquisition of marine proteins" or "Animal feed" or "Automated data collection" or ("bioactive compounds" and collagen or gelatin* or biopolymers or chitin or chitosan or hydroxyapatite or carotenoids or pigments or proteins or "protein hydrol*" or enzymes or proteinases or pepsin or trypsin or chymotrypsin or collagenases or chitinases or chitosanases or peptides or "amino acids" or gelatine or "gelatinous solutions" or collagen or minerals or "polyunsaturated fatty acids" or oils or vitamins or

polyphenol* or carotenoids or astaxanthin or "calcium phosphate" or taurine or creatine) or (Bioactiv* and "ability to stimulate fibroblast production" or "accelerate absorption of dietary calcium" or anticoagulant or antiplatelet or antihypertensive or antimetastatic or antioxidant or anti-inflammatory or antiproliferative or anticancer or antithrombotic or anti-stroke or pro-inflammatory or immunomodulating or pro-angiogenic or cardioprotective or neuroprotective or antiadipogenic or angiogenic or antiarrhythmic* or antiproliferative or "apoptotic effects" or "lipid-lowering function" or brain-boosting or condition* or moisturi* or emollient or "nourishment of scalp hair" or "treatment of osteoporosis" or "treatment for brittle nails" or "catalytic activity" or "fat scavenger*" or "inhibition of Beta-secretase activity" or "inhibitor of angiotensin converting enzyme" or wound-healing or "cartilage matrix synthesis") or biocompatibility or amphiphilicity or non-toxicity or biodegradable or "Biofuel production" or "biogas production" or "Biomedical applications" or "carotenoprotein production" or "dyeing agent" or "colorant agent" or cosmetics or "personal care" or "Critical control point" or Decontamination or demineralization or deproteinization or "Designer Foods" or "supplement* Foods" or "function* Foods" or "drug carrier" or "drug delivery" or "drug development" or "efficient production" or "quality production" or "Exploitation of biomaterials" or "gene delivery" or "increas* shelf life" or "increase* calcium bioavailability" or "maintenance of bone integrity" or "maintenance of skin homeostasis" or "Marine Ingredients" or "natural bioactive materials" or "New distribution patterns" or "strategic management" or nutraceutical or healthcare or "nutritional enrichment" or "nutritional quality" or pharmac* or "Preservation technologies" or "production of PUFA-enriched oils" or "Protein feedstuff aquaculture diets" or "Raw materials" or "resource utilization" or "skin health-promoting" or "specialized products" or "bone substitution materials" or "supplements in human nutrition" or "Sustainable fish* management" or "Sustainable resource development" or "valorization routes").
